# Jenner-predict server: prediction of protein vaccine candidates (PVCs) in bacteria based on host-pathogen interactions

**DOI:** 10.1186/1471-2105-14-211

**Published:** 2013-07-01

**Authors:** Varun Jaiswal, Sree Krishna Chanumolu, Ankit Gupta, Rajinder S Chauhan, Chittaranjan Rout

**Affiliations:** 1 Department of Biotechnology and Bioinformatics, Jaypee University of Information Technology, Waknaghat, Solan, Himachal Pradesh 173234, India

**Keywords:** Protein vaccine candidates (PVCs), Host-pathogen interactions, Domain, Antigen, Reverse vaccinology, Virulence

## Abstract

**Background:**

Subunit vaccines based on recombinant proteins have been effective in preventing infectious diseases and are expected to meet the demands of future vaccine development. Computational approach, especially reverse vaccinology (RV) method has enormous potential for identification of protein vaccine candidates (PVCs) from a proteome. The existing protective antigen prediction software and web servers have low prediction accuracy leading to limited applications for vaccine development. Besides machine learning techniques, those software and web servers have considered only protein’s adhesin-likeliness as criterion for identification of PVCs. Several non-adhesin functional classes of proteins involved in host-pathogen interactions and pathogenesis are known to provide protection against bacterial infections. Therefore, knowledge of bacterial pathogenesis has potential to identify PVCs.

**Results:**

A web server, Jenner-Predict, has been developed for prediction of PVCs from proteomes of bacterial pathogens. The web server targets host-pathogen interactions and pathogenesis by considering known functional domains from protein classes such as adhesin, virulence, invasin, porin, flagellin, colonization, toxin, choline-binding, penicillin-binding, transferring-binding, fibronectin-binding and solute-binding. It predicts non-cytosolic proteins containing above domains as PVCs. It also provides vaccine potential of PVCs in terms of their possible immunogenicity by comparing with experimentally known IEDB epitopes, absence of autoimmunity and conservation in different strains. Predicted PVCs are prioritized so that only few prospective PVCs could be validated experimentally. The performance of web server was evaluated against known protective antigens from diverse classes of bacteria reported in Protegen database and datasets used for VaxiJen server development. The web server efficiently predicted known vaccine candidates reported from *Streptococcus pneumoniae* and *Escherichia coli* proteomes. The Jenner-Predict server outperformed NERVE, Vaxign and VaxiJen methods. It has sensitivity of 0.774 and 0.711 for Protegen and VaxiJen dataset, respectively while specificity of 0.940 has been obtained for the latter dataset.

**Conclusions:**

Better prediction accuracy of Jenner-Predict web server signifies that domains involved in host-pathogen interactions and pathogenesis are better criteria for prediction of PVCs. The web server has successfully predicted maximum known PVCs belonging to different functional classes. Jenner-Predict server is freely accessible at http://117.211.115.67/vaccine/home.html

## Background

*In silico* prediction has been proved to be of great significance among various disciplines of life sciences including biomedical research [1]. The conventional vaccine development methods are time consuming as they require cultivation of pathogenic microorganisms in laboratory conditions and their dissection using microbiological, biochemical and immunological methods in order to identify the components important for immunogenecity. These methods are ineffective in circumstances where the cultivation of bacteria is difficult or impossible. The other limitations arise when the expression of protective antigens is less or absent in *in vitro* conditions compared to *in vivo* diseased conditions [2]. In comparison to conventional live attenuated vaccines, subunit vaccines are more reliable as far as safety is concerned [3]. Vaccine candidate identification is an essential and important component in subunit vaccine development. The integration of genomics in vaccine research (vaccinogenomics) is expected to revolutionize novel vaccine candidate identification [4]. Computational approach, especially reverse vaccinology (RV) method assists the identification of vaccine candidates from genomes without culturing microorganisms and thus facilitates the subunit vaccine development. These methods are useful in reducing time, cost and number of wet lab experiments [2].

The RV is a computational pipeline for identification of vaccine candidates against microorganisms from their genome sequences. Thus, all proteins of an organism can be screened computationally for their vaccine potential. Significant success of this principle for vaccine development had already been demonstrated in several pathogens, including *Neisseria meningitides*[5], *Helicobacter pylori*[6], *Streptococcus pneumoniae*[7], *Porphyromonas gingivalis*[8], *Chlamydia pneumoniae*[9] and *Bacillus anthracis*[10]. The relevance of this method was recognized when vaccines developed from capsular polysaccharides of *N. meningitides* B failed due to cross reactivity against human tissue [5]. Application of RV techniques for PVC identification and then *in vivo* testing led to the development of licensed broad specificity protein vaccine, 5CVMB, against *N. meningitides*. This vaccine contains 5 protein antigen components, GNA2132, GNA1870, GNA1030, GNA2091 and NadA, which were primarily discovered by RV methods [11]. However in earlier RV techniques, protein localization (secretory, outer-membrane, transporter or others) was used as the main criterion for identification of PVCs. As a result, a large number of proteins were required to be expressed, purified and tested to obtain few vaccine candidates leading to enormous loss of cost and time.

On the other hand, identifying immunogenic proteins (PVCs) by using epitope prediction software and web servers have several limitations. Comparative studies have shown that B-cell epitopes (BCEs) and class-II MHC-binding T-cell epitopes (TCEs) prediction methods are not accurate [12-15]. Over-prediction, inability in exact position prediction of epitopes and absence of success in identifying known epitopes in proteins are major concerns in vaccine candidate identification. Until now the available PVCs prediction software and web servers have not been much effective for identification of vaccine candidates from genomes for vaccine design. VaxiJen server, based on discriminant analysis and partial least square (DA-PLS) methods, was developed by using datasets of known (positive) protective antigenic and non-antigenic (negative) proteins to predict PVCs [16]. Surprisingly, it predicts more than half of proteins from a given bacterial proteome as protective antigens with default parameters making its usage almost impractical. Further, existing software and web servers predict different proteins as vaccine candidates from same proteome sequences. For example, different proteins were predicted from *S. pneumoniae* proteome by VaxiJen [16] server and new enhanced reverse vaccinology environment (NERVE) [17] software. From 2202 proteins of *S. pneumoniae,* VaxiJen (with cut-off of 0.6) and NERVE predicted 313 and 58 as PVCs, respectively while only 20 proteins were common between them. None of the common PVCs matched with 18 known vaccine candidates in *S. pneumoniae* (Additional file 1: Table S1). This outcome complicates decision process regarding which tool’s output should be taken for experimental testing to identify vaccine candidates.

The method used in NERVE [17] and Vaxign [18] tools presumed that extracellular proteins having adhesin-likeliness are potential vaccine candidates. Although adhesin-likeliness of a protein is an important criterion, it should not be considered as the only one because several non-adhesin functional classes of proteins (*i.e.* invasin, porin, flaggelin, etc.) are also involved in host-pathogen interactions or pathogenesis and many of them are known to be antigenic [19-31]. It has been suggested that targeting host-pathogen interactions and disease processes at molecular level can be used for novel vaccine discovery [4]. In several cases, the immune responses against these non-adhesins were known to provide protection against microbial infection [19-31]. Invasin, porin, flagellin and toxin have roles in host cell invasion [32]; transportation activity is associated with pathogenesis and virulence [33]; chemotaxis, adhesion and colonization are making pathogenic bacteria to be virulent [34]; and host cell death [35], respectively. Bacterial fibronectin-binding proteins (FBPs) target host fibronectin for adhesion and colonization [36]; transferrin-binding proteins (TBP) are used by bacteria to obtain iron directly from host transferrins [37]; and penicillin-binding proteins (PBPs) are involved in peptidoglycan biosynthesis to maintain cell wall structure and protection [38]. The solute binding proteins (SBPs) are used to capture nutrients like iron to overcome the environment devoid of free nutrients within the host [21]. Choline-binding proteins (CBPs) in some bacteria perform adhesin-like function [39]. Functional classes of proteins involved in virulence [26], invasion [23] and colonization [29]; porins [22] and flagellin [28]; and binding proteins of choline [20], penicillin [27], transferrin [25], fibronectin [24] and solute [21] are important in host-pathogen interactions and pathogenesis. Since many proteins from these functional classes provide protective immune responses against microbial infection [19-31], the knowledge of host-pathogen interactions related to bacterial pathogenesis could be used to rationalize and improve vaccine candidate prediction.

**Figure 1:**
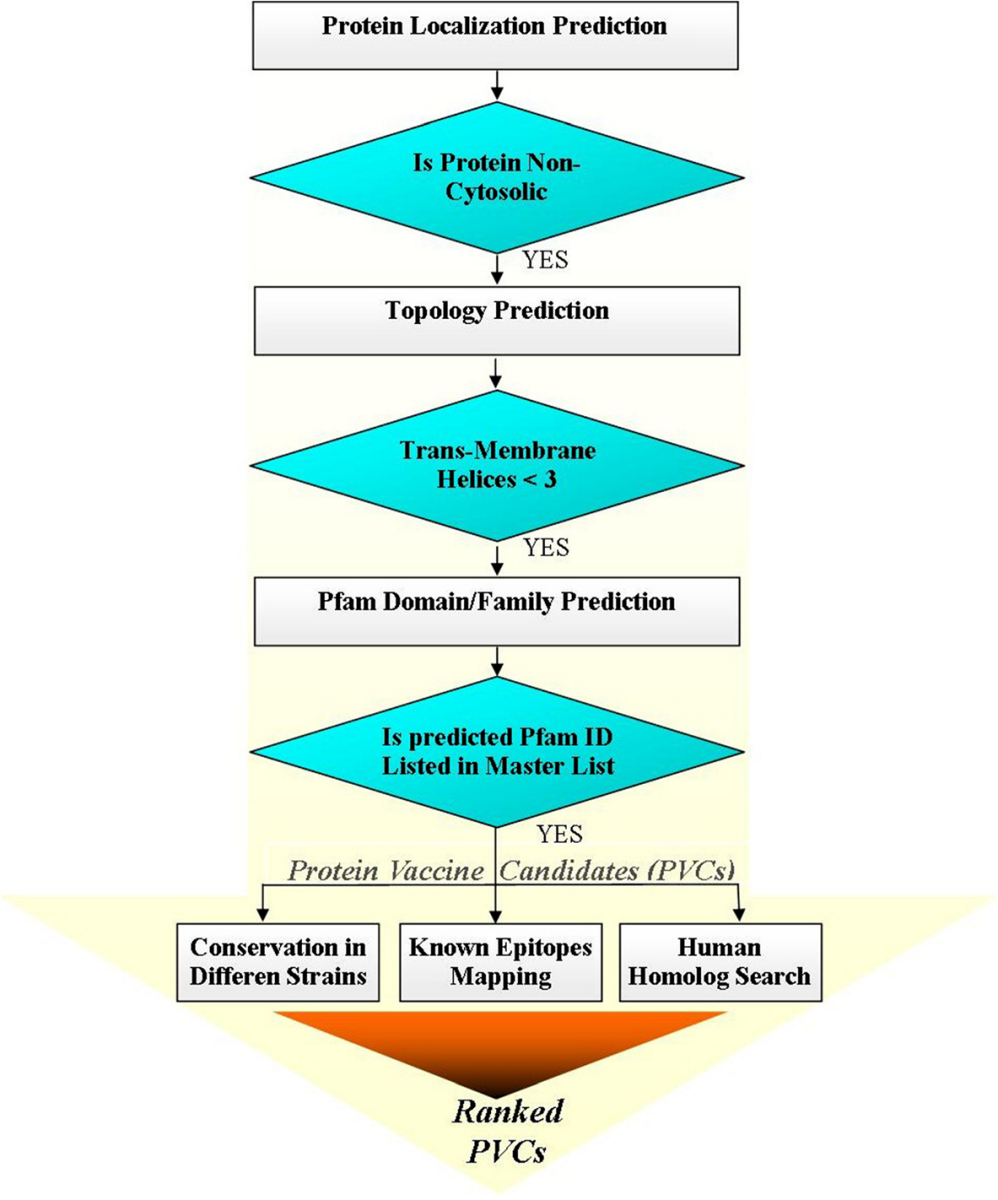
Flow chart depicting pipeline of Jenner-Predict server.

A web server, Jenner-Predict, has been developed which is capable of predicting PVCs from proteome/protein sequences. It is based on the principle that non-cytosolic proteins having functions (domains) important in host-pathogen interactions and/or pathogenesis are potential vaccine candidates (Figure 1). It has two broad components: PVCs prediction and analysis of their vaccine potential. The PVCs prediction is performed in three sequential steps: prediction of subcellular localization, expressibility in laboratory and presence of domains critical in host-pathogen interactions and pathogenesis. Software PSORTb 3.0 is used for protein subcellular localization prediction [40]. A protein has high probability of failure to express in experiment [5] when it has more trans-membrane helices. HMMTOP 2.0 [41] software is used for topology prediction and proteins with more than two trans-membrane helices are discarded. Proteins pass through above two filters, and having domains involved in host-pathogen interactions and pathogenesis from functional classes of adhesin, invasin, toxin, porins, colonization, virulence, flagellin, penicillin-binding, choline-binding transferring-binding, fibronectin-binding and solute-binding proteins are selected as vaccine candidates. Standalone Pfam sequence search is used for prediction of domains [42]. Vaccine potential of PVCs is predicted on the basis of their possible immunogenicity, absence of autoimmunity, and conservation across different pathogenic and non-pathogenic strains of same bacteria. Known BCEs and TCEs from immune epitope database (IEDB) [43] are mapped separately on predicted PVCs to know their possible immunogenic region and potential. This mapping of antigenic determinant (epitope) is instrumental in predicting humoral (BCE) or cellular (TCE) or both immune responses of PVC. Since PVCs specific to pathogenic strains are expected to be involved in virulence [26], therefore conservation of PVCs in different pathogenic strains of same organism is determined to provide more robust vaccine candidates. The PVCs having homolog(s) in host (human) are provided by the web server. Such PVCs may produce autoimmunity [44] or less immune response [45]. Taking into account above criteria, output of the web server is provided as prioritized PVCs in the result table. Comparison among PVC prediction methods has shown that Jenner-Predict server’s performance is better.

## Results

The web server, Jenner-Predict, has been developed to predict PVCs from proteome or protein(s) sequences for subunit vaccine development on the basis of domains critical to host-pathogen interactions and pathogenesis. Besides predicting PVCs, it also furnishes information crucial to determine their vaccine capability in terms of immunogenic potential by matching PVCs against IEDB epitopes, autoimmunity through matching PVCs with human proteome, and their conservation across different pathogenic and non-pathogenic strains of the organism (Additional file 2: Figure S1). A tutorial explaining how to submit a job as well as user-friendly interpretation of results is available at the web server’s home page. The web server gives higher priority to PVCs containing more IEDB epitope matches as they increase their possibility to be immunogenic. The PVCs containing exact IEDB epitopes match are shown in white background. The web server also decreases priority of PVCs having human homologs as such PVCs should be discouraged from further vaccine development process. This prioritization is instrumental in selecting few PVCs for further vaccine development experiments. The performance of the web server was evaluated against reported vaccine candidates in *S. pneumoniae* (gram positive) and *E. coli* (gram negative), proteins {both positive (protective antigen) and negative (non-antigen)} used for the development of VaxiJen server [16] and protective antigens from more than 40 bacteria reported in Protegen database [46].

### PVCs Prediction in *S. pneumoniae* and *E. Coli*

In *S. pneumoniae* proteome, Jenner-Predict server predicted 69 proteins as vaccine candidates (Additional file 3: Table X1). As VaxiJen server predicts more than half of a proteome as vaccine candidates in any bacteria with default VaxiJen probability score, 0.4, a cut-off of 0.6 was considered to restrict the number of PVCs so that the performance of this approach can be compared against all other methods. From *S. pneumoniae* proteome, our web server predicted 10 out of 18 known non-cytoplasmic PVCs whereas the software, NERVE, and servers, Vaxign and VaxiJen, predicted only 7, 6 and 3 PVCs, respectively (Additional file 1: Table S1). As compared to other methods, the PVCs predicted exclusively by Jenner-Predict server were STK [47], NanA [23], and PsaA [48] that are having PASTA, BNR, and SBP domains, respectively (Additional file 1: Table S1). Other than 10 reported vaccine candidates from 69 PVCs (Additional file 3: Table X1), our web server predicted 18 ABC-transporters and solute-binding, 6 choline-binding, 4 penicillin-binding, 2 LysM-containing domain, 3 cell wall anchors, etc. ABC-transporter [49], choline-binding [20] and penicillin-binding [27] proteins are known to be potential PVCs in different bacteria including *S. pneumoniae*.

In *E. coli* proteome, Jenner-Predict server predicted 253 proteins as vaccine candidates (Additional file 4: Table X2) whereas the NERVE and VaxiJen predicted more than 500 and 280 proteins, respectively. Our web server predicted 23 out of 28 known PVCs whereas software, NERVE and servers, Vaxign and VaxiJen, predicted 21, 18 and 21 PVCs, respectively. The PVCs missed out by other methods due to being non-adhesins were OmpA (outer-membrane protein A), BtuB (cobalamin outer-membrane transporter), TolC (channel protein) and IreA (putative iron-regulated outer membrane virulence proteins) (Additional file 5: Table S2). Besides 23 known protective antigens, the majority of predicted PVCs by our web server were 51 BPD transporter proteins, 32 solute-binding proteins [21] and 32 fimbrial proteins [50] (Additional file 4: Table X2).

### Prediction of PVCs against protegen database and datasets used in VaxiJen server development

The results of our web server for prediction of known protective vaccine candidates from more than 40 diverse bacteria reported in Protegen database and its comparison against other similar methods has been presented in Additional file 6: Table S3. Our web server predicted 137 out of 177 protective antigens (Refer ‘Collection of data for web server validation’ subsection in Methods) from Protegen database whereas software, NERVE, and servers, Vaxign and VaxiJen, predicted 121, 89 and 97, respectively. The protective antigens (PAs) which were only predicted by our method and skipped by others (NERVE, Vaxign and VaxiJen) belong to functional classes of solute binding, toxin, invasin, etc. (Additional file 6: Table S3). The Jenner-Predict server was found to be efficient in discriminating between antigens and non-antigens. From the 83 PAs (positive dataset) used in VaxiJen server development, NERVE, Vaxign and VaxiJen predicted 53, 47 and 46 proteins, respectively whereas our web server predicted 59 PVCs (Additional file 7: Table S4 (A)). From negative dataset (considered as non-antigens) of 33 proteins, the NERVE, Vaxign and VaxiJen methods predicted 8, 5 and 3 proteins to be vaccine candidates, respectively compared to 2 proteins (Q48919 and Q53247) by our web server (Additional file 7: Table S4 (B)). Negative dataset proteins were considered as non-antigens. But one out of two PVCs predicted by our web server has already been known to be antigenic: fibronectin-attachment protein (Q48919) provides protective immunity against *Mycobacterium avium* infection [51].

### Validation of Jenner-predict

Sensitivity and specificity indices of different PVC prediction methods have been presented in Table 1. Jenner-Predict server’s PVCs prediction accuracy is better at all levels: bacterial proteomes, Protegen database and datasets used for VaxiJen server development. Unavailability of total number of known vaccine candidates in a proteome prevented us to calculate sensitivity and specificity values for proteome sequences. The results of PVC prediction from two proteomes by different methods have been provided in Table 1. Detailed comparison of results w.r.t known vaccine candidates of *S. pneumoniae* and *E. coli* by different methods has been provided in Additional file 1: Table S1 and Additional file 5: S2, respectively. On dataset used in development of VaxiJen server, sensitivity and specificity of our tool were 0.711 and 0.940, respectively whereas comparable methods NERVE, Vaxign and VaxiJen have corresponding values 0.639 and 0.765; 0.494 and 0.853 and 0.554 and 0.909, respectively. For the Protegen database, only sensitivity was calculated as specificity calculation was not feasible due to lack of negative dataset. The sensitivities of NERVE, Vaxign and VaxiJen were 0.684, 0.491 and 0.548, respectively as compared to 0.774 for Jenner-Predict server.

**Table 1:** Performance evaluation of Jenner-Predict server against existing software, NERVE, and web servers, Vaxign and VaxiJen^*^

**S. No.**	**Different software and web servers**	**Prediction in**	**Prediction in**	^**$**^**Protegen (bacterial)**		^**%**^**Data used for VaxiJen server development**		
		***S. pneumoniae***^***#***^	***E. coli***^***#***^	**No of predictions**	**Sensitivity**	**Positive data**	**Negative data**	**Sensitivity (Specificity)**
1.	NERVE	**7** (18) [58]	**21** (28) [527]	121 (177)	0.684	53 (83)	8 (33)	0.639 (0.758)
2.	Vaxign	**6** (18) [61]	**18** (28) [286]	89 (177)	0.502	41 (83)	5 (33)	0.494 (0.848)
3.	VaxiJen	**3** (18) [313]	**21** (28) [955]	97 (177)	0.548	46 (83)	3 (33)	0.554 (0.909)
4.	Jenner-Predict	**10** (18) [69]	**23** (28) [253]	137 (177)	0.774	59 (83)	2 (33)	0.711 (0.940)

^*^ See method section for details.

^*#*^ Values within square bracket indicates total number of proteins vaccine candidates (PVCs) predicted by respective software/server. Value within parenthesis indicates experimentally known protective vaccine candidates in that organism whereas the values in bold give the number of PVCs predicted by respective software/server from experimentally known protective vaccine candidates in that organism.

^$^ Values indicate the number of PVCs predicted by respective software/server from 177 bacterial protective antigens (PAs) taken for evaluation.

^%^ Values indicate the number of PVCs predicted by respective software/server from 83 and 33 proteins for positive and negative datasets, respectively.

## Discussion

The motivation behind developing ‘Jenner-Predict’ web server is to provide credible vaccine candidates and information regarding their vaccine potential in terms of possible immunogenicity, absence of autoimmunity and conservation so that subunit vaccine development can be accelerated. The outcome of the web server has substantiated that domains involved in host-pathogen interactions are better criterion for prediction of PVCs than approaches dependent upon only adhesin-likeliness or machine learning. As PVCs are predicted based on their functions, biologists can assess the importance of a given function in pathogenesis for that organism. The information regarding the function of PVC could be instrumental for vaccine development. For example, colonization is crucial in *Streptococcus* pathogenesis and proteins (also predicted by Jenner-Predict) involved in this process were used as vaccine candidates [52].

Most of the earlier RV methods focused on outer membrane or secretory proteins of a proteome to identify PVCs. Pizza *et al.* screened proteome sequences of *N. meningitis* to identify proteins which were probably surface exposed or involved in transportation and obtained 570 proteins. Out of them, 350 proteins were expressed and experimentally tested for their immunogenic potential. Finally, 7 proteins were found to provide protective immunity against *N. meningitides*[5]. Similarly, Wizemann *et al.* searched for motifs related to secretory or surface binding proteins in *S. pneumoniae* proteome and identified 130 proteins containing such motifs. Out of them, 108 were expressed and tested for their protective immunity. Finally, 6 proteins were found as protective antigens. Similar studies were performed for *P. gingivalis*[8] and *C. pneumoniae*[9]. Although proteins providing immunity were identified in these studies but the number of experiments, cost and time requirement were enormous even for identifying PVCs from a particular localization. On the contrary, Jenner-Predict server is relying on protein domains involved in host-pathogen interactions for providing reasonably less number of prioritized vaccine candidates from a proteome. For better validation of vaccine candidates, the user may select few prospective vaccine candidates for experimental testing to verify their protective immunity.

The BCE and TCE mapping algorithms were developed to identify possible immunogenic region(s) and consequently prediction of immunogenic potential of a protein. But these methods have drawbacks of over-prediction and even predict epitope(s) in known non-antigenic proteins [12-15]. Currently available antigen or PVC prediction methods were not validated on complete or diverse data. NERVE software was evaluated on its prediction ability of popular vaccine candidates from five bacterial proteomes instead of all known vaccine candidates in those organisms. Similarly, the Vaxign server was evaluated against only limited number of known outer membrane protein (OMP) vaccine candidates from uropathogenic *E. coli*[18]. Further, the VaxiJen server was developed on limited data. Even some of the sequences used as the negative data (non-antigenic) for web server development were predicted as vaccine candidates (antigenic proteins) by all other methods (Additional file 7: Table S4 (B)). Our web server predicted two such proteins (Q48919 and Q53247) as PVCs from the negative dataset sequences. Experimental data confirmed that alanine and proline rich secreted protein (Q48919) is immunogenic [51] whereas the other protein (Q53247) is a periplasmic serine or membrane protease (htrA gene) and has already been reported as protective antigen in *Haemophilus influenzae*[53]. The other PVCs predicted from negative dataset by NERVE, Vaxign and VaxiJen have no evidence of being immunogenic. This outcome justifies higher sensitivity of our method.

To provide prospective PVCs of a proteome, the predicted vaccine candidates are prioritized by Jenner-Predict server. The PVCs having more IEDB epitope matches are ranked higher as such epitope match increases their possibility to be immunogenic. Epitopes identified using ‘hands on’ peptide-by-peptide *in vitro* assays as in case of immunoepitope database have been more substantive than epitopes predicted by using *in silico* methods. Therefore, known and validated epitopes from the IEDB [43] are mapped on PVCs to predict potential immunogenic regions. The Jenner-Predict server de-prioritizes PVC having human homolog(s) as they can potentially cause autoimmunity [44] or produce low immune response [45]. Conservation information of PVCs is provided by the web server to demonstrate their broad specificities. Since the web server provides conserved and potential immunogenic PVCs, it may be useful to replace the existing strain-specific vaccine candidates. For example, the established vaccine candidate, PspA is having choline-binding protein (CBP) domain and it has limited application from vaccine point of view as it is strain-specific. In contrast, our tool predicted cbpE (gi|225858728) protein which is conserved across different strains of *S. pneumoniae* and has the same CBP domain. Since this protein is surface exposed, and involved in nasopharyngeal colonization and/or dissemination of *S. pneumoniae* which is important for virulence, this protein may further be explored for vaccine development process [39].

Jenner-Predict server predicted 3 experimentally known promising PVCs, STK [47], NanA [23], and PsaA [48] in *S. pneumoniae* which are containing domains from non-adhesins such as PASTA, BNR and SBP, respectively (Additional file 1: Table S1) and they are known to be immunogenic. Similarly, our method predicted established non-adhesin vaccine candidates, Omp A, IreA, BtuB and TolC (provides protective immune responses [54] in *E. coli*). The wide-ranging applicability of the web server to all bacteria is substantiated by its high sensitivity for predicting diverse protective antigens from more than 40 pathogenic bacteria reported in Protegen database (Additional file 6: Table S3) and dataset used for VaxiJen server development (Additional file 7: Table S4). Our domain based method was effective in predicting many established non-adhesin vaccine candidates reported in Protegen database [46] such as 13 toxins, 12 binding proteins (fibronectin, penicillin, choline, etc.), 10 membrane proteins, 6 surface proteins, etc. (Additional file 6: Table S3) which were not predicted by other methods. These protective antigens are involved in many important pathogenesis processes like virulence, invasion, colonization, iron acquisition, osmo-regulation, etc. (Additional file 6: Table S3). The sensitivity of the web server was further substantiated by its prediction of immunogenic protein, Q48919, from negative dataset used for training of VaxiJen server (Additional file 7: Table S4 (B)). Higher sensitivity and specificity of Jenner-Predict server (Table 1) justifies the domains involved in host-pathogen interactions and pathogenesis are better criteria for PVCs prediction than other existing approaches.

## Conclusions

The Jenner-Predict server has been developed to predict potential PVCs and to provide their vaccine potential with an objective of assisting subunit vaccine development. The web server was validated on independent and diverse datasets, where it outperformed other PVC prediction tools. Its performance substantiated that the proteins involved in host-pathogen interactions and pathogenesis are better criteria than methods based on machine learning or adhesin-likeliness. Our method predicts less number of proteins with high prediction accuracy which confirms its reliability. Mapping of known epitopes from IEDB database on PVCs increases the probability of a protein to be immunogenic. Comparison of these PVCs with human proteome sequences reduces the chance of their failure due to autoimmunity. Conservation of PVCs in pathogenic strains provides crucial information on their broad-specificities. The web server demonstrated that domain-based method can be used to predict PVCs from pathogen proteomes. Since the web server provides prioritized PVCs, few prospective proteins from a proteome could be taken for experimental evaluation to identify subunit vaccine candidates.

## Methods

### Data collection and generation

Proteomes of all bacteria were downloaded from NCBI ftp (http://ftp.ncbi.nih.gov/genomes/Bacteria/all.faa.tar.gz). The proteomes of *S. pneumoniae* strain 70585 and *Escherichia coli* uropathogenic strain CFT073 were collected from above proteomes. Human proteome sequences were downloaded from the EBI ftp site (http://ftp.ebi.ac.uk/pub/databases/integr8/fasta/proteomes) for prediction of human homologs in PVCs. For the development of web server, standalone version of four softwares (Additional file 2: Figure S1), NCBI BLAST (http://ftp.ncbi.nlm.nih.gov/blast/executables/blast+/LATEST/), PSORTb 3.0 (http://www.psort.org/psortb/), HMMTOP 2.0 (http://www.enzim.hu/hmmtop/) and HMMER 3.0 (http://hmmer.janelia.org/) were downloaded from their respective websites. PSORTb 3.0 predicts subcellular localization of a given protein sequence based on its amino acid composition, similarity to proteins of known localization, and presence of different motifs and signal peptides [40]. HMMTOP software uses hidden Markov model (HMM) to predict transmembrane helices based on the difference in the amino acid distributions in various structural parts of proteins [41]. For prediction of domains in protein sequences, Perl program, pfam_scan.pl and Pfam library of HMMs for protein families were downloaded from Pfam website (http://pfam.janelia.org/).

For prediction of immunogenic regions in PVCs, experimentally known immunogenic epitope sequences of all T-cell epitope (TCE) and B-cell epitope (BCE) assays were downloaded from IEDB (http://www.iedb.org/) in CSV format. Peptide epitope with literature reference, epitope ID, GI of source protein, and source and host organism’s information were extracted from these TCEs and BCEs assays. In case of TCEs, MHCs allele names were also extracted. All epitope sequences were stored in ‘fasta’ format for comparison against PVCs. For discontinuous BCEs, corresponding protein sequences were downloaded from database, and stretch of continuous sub-part protein sequences containing all the residues of discontinuous epitope positions was extracted. These subsequences (epitopes) were also stored in ‘fasta’ format for comparison against predicted PVCs.

### Collection of data for web server validation

Experimentally known protective antigens were collected from four diverse sources to evaluate the performance of Jenner-Predict server against existing methods. Known non-cytosolic protective PVCs from the two pathogenic bacteria, *S. pneumoniae* (gram-positive) and *E. coli* (gram-negative) were collected from literature. Different experiments had identified 18 and 28 non-cytosolic proteins to be protective antigens for *S. pneumoniae* and *E. coli*, respectively (Additional file 1: Table S1 and Additional file 5: Table S2). To demonstrate effectiveness of the web server in predicting vaccine candidates across bacteria, non-cytosolic protective antigens sequences reported in ‘Protegen’ database [46] were retrieved for evaluation as well. Out of the 257 reported bacterial protective PVCs in Protegen database, 211 were predicted to be non-cytosolic by PSORTb 3.0. After removing 11 antigens having more than 2 trans-membrane helices and sequences which are 90 percent identical among themselves by using CD-HIT (http://weizhong-lab.ucsd.edu/cd-hit/ref.php), 177 bacterial protective PVCs were selected for evaluation. In addition to above, non-cytosolic proteins from datasets used for VaxiJen [16] server development were also taken for evaluation. Positive and negative training and test datasets containing 100 sequences of each in the form of Swiss-Prot IDs were collected and then their sequences were retrieved. PSORTb was used to predict their localization and only non-cytosolic proteins were retained. Finally, 83 and 33 non-cytosolic positive (protective antigen) and negative (non-antigen) sequences were selected for comparison of performances. The sequences used for validation in both Protegen and VaxiJen datasets are highly diverse (more than 90% sequences are less than 40% identical).

### Server architecture

The web server comprised of a client interface and a main application program. The client interface was developed using HTML language which takes input either in the form of protein sequence(s) in fasta format or a proteome of listed bacteria. The submitted fasta sequence(s)/proteome are processed by the in-house backend Perl-CGI script which posts information provided by the user to the main application program in a queue. This Perl-CGI script generates an URL link where the status information or output of a given job will be available. The self developed programs and other available standalone software (Figure 1) are used by ‘main application’ program for the analysis of protein sequences one after another to predict PVCs. The main application program also provides the output table as prioritized PVCs.

### Pfam domain identification

Domains are basic building blocks of proteins. Searching of a protein sequence against Pfam library of HMMs enables to find domain architecture present in that protein [42]. The Pfam has been used in several genome projects including human for large scale functional annotation of genomic data [55]. A list called, ‘Master list’, was prepared which contains Pfam IDs (domain) from the functional classes of proteins involved in host-pathogen interactions and pathogenesis [19-28]. For preparing the list, Pfam database was subjected to text search with individual key words ‘adhesin’, ‘choline binding protein’, ‘bacterial extracellular solute-binding protein’, ‘porin’, ‘invasin’, ‘fibronectin-binding protein’, ‘transferrin-binding protein’, ‘virulence’, ‘penicillin-binding protein’, ‘flagellin’, ‘colonization’, ‘host-pathogen interaction’ and ‘toxin’ to identify domains from each classes of proteins. Then all hits of domains from each keyword were manually checked for their possible role in host-pathogen interactions. Only those families/domains were included in the ‘Master list’ which have significant functional role in host-pathogen interactions and/or pathogenesis (Table 2). This ‘Master list’ of domains was used for the prediction of PVCs from non-cytosolic proteins.

**Figure 2:**
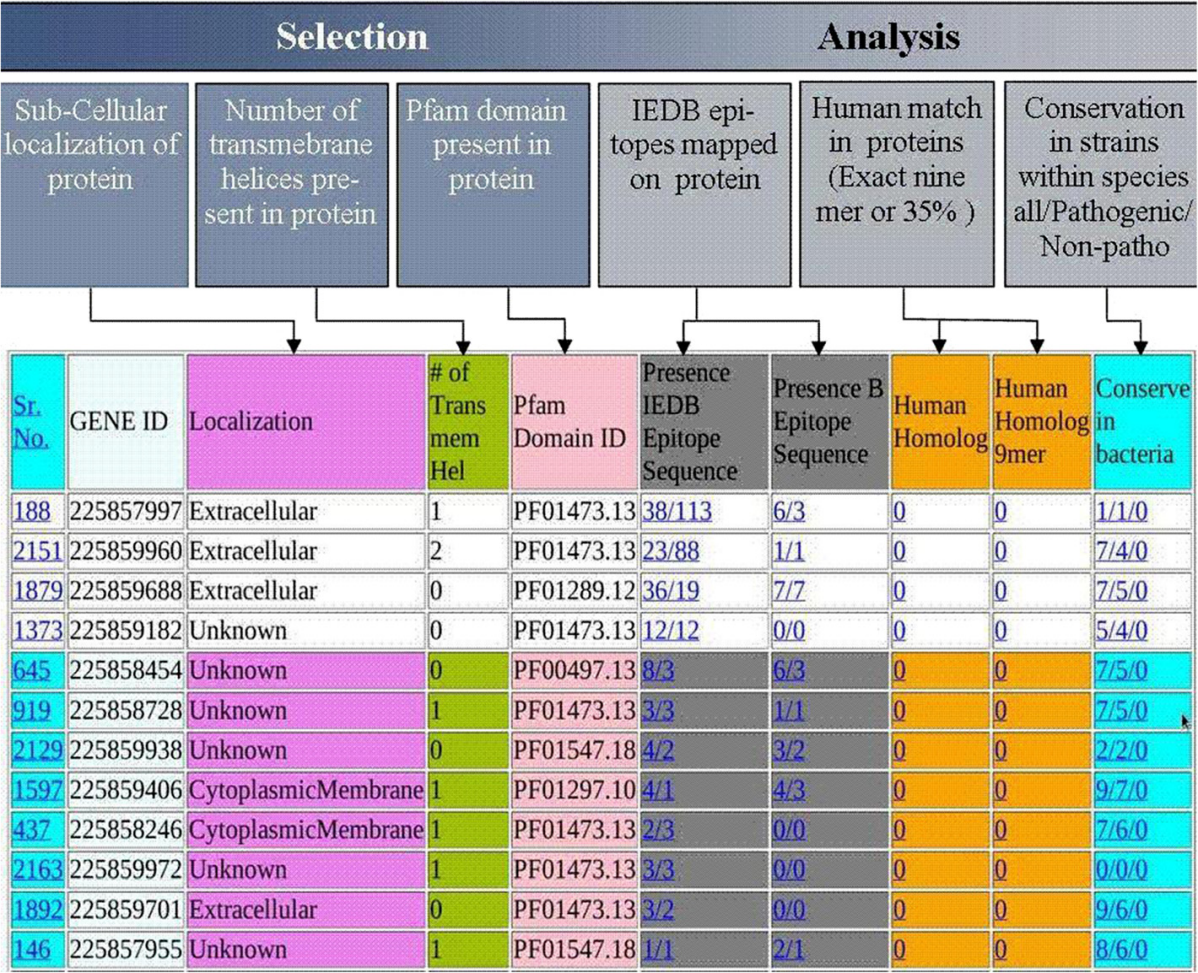
Sample output of Jenner-Predict server.

### Implementation

The server, Jenner-Predict, has two major components: PVCs prediction and analysis of their vaccine potential (Figure 1 and Additional file 1: Figure S1). In first component, software PSORTb 3.0 [40] and HMMTOP 2.0 [41] are used to predict subcellular localization and number of transmembrane helices, respectively. The former discards cytoplasmic proteins whereas the latter rejects proteins having more than two transmembrane helices [5]. Proteins passing through the above two filters are then subjected to Pfam domain/family search to determine their domains. Finally, role of identified domains in host pathogen interaction and pathogenesis is checked according to its presence in ‘Master list’ (described in ‘Pfam Domain Identification’ subsection and Table 2). Proteins having domains/families matching with Pfam domains/families listed in the ‘Master list’ are selected as PVCs.

**Table 2:** Key words used and selection of Pfam domains for protein vaccine candidate prediction

**Sr. No.**	**Key word used for PFam domain search**	**No of domain hits**	No of selected domains^*^	**Reference**
1.	Adhesin	166	96	19
2.	Choline binding protein	29	12	20
3.	Bacterial extracellular solute-binding protein	36	8	21
4.	Porin	66	46	22
5.	Invasin	30	25	23
6.	Fibronectin-binding protein	50	25	24
7.	Transferrin-binding protein	24	6	25
8.	Virulence	402	145	26
9.	Penicillin-binding Protein	14	8	27
10.	Flagellin	22	12	28
11.	Colonization	23	14	29
12.	Host-pathogen interaction	9	4	30
13.	Toxin	542	110	31

^*^Only those families/domains were included which are involved in host-pathogen interactions and/or pathogenesis.

In second component, vaccine potential of the predicted PVCs’ is performed by taking three different measures into account (Figure 1): immunogenicity, autoimmunity and conservation. Immunogenic potential (putative immunogenic regions in terms of BCEs and TCEs) of PVCs is predicted by exactly matching of IEDB epitopes (Refer ‘data collection and generation’ subsection) against the PVCs by using standalone BLAST with minimum matching length of 9 [56] and 80% identity cut-off. For autoimmunity prediction, the BLAST is used to find similarity between PVCs and human proteins by two different methods: i) cut-off of 35% identity in at least 80 amino acids length of PVC [57], and ii) continuous identical matching of 9 or more positions in the alignment [56]. BLAST is also used to identify conservation of PVCs in different pathogenic strains of a given organism. The PVC is compared against different strains of the same organism with a cut-off greater than 85 percent sequence identity with minimum of 90% query coverage. To determine conservation of PVC in pathogenic and non-pathogenic strains separately, names of pathogenic and non-pathogenic strains of each organism are stored in two separate flat files under each category. Information on pathogenic or non-pathogenic strains of each individual organism is extracted from the respective files.

### Output

The server, Jenner-Predict, has been designed for easy submission of a job as well as user-friendly interpretation of results. Just after job submission, an URL link is generated which the user may bookmark for tracking the jobs status which is processed in a queue. Once a job has been completed, the output is provided in a tabular format and a sample output is represented in Figure 2. The information provided in different columns are as follows: 1. Sr. No.; 2. Gene Id; 3. Localization; 4. No. of transmembrane helices; 5. Pfam domain ID; 6. No. of IEDB TCE(s) match(s); 7. No. of IEDB BCE(s) match(s) (Hyperlinks on 6 and 7 showing details of matching epitopes); 8. and 9. Autoimmunity information through 35% identical matches in 80 AA lengths, and No. of continuous 9-mer identical match in an alignment, respectively; and 10. Conservation in number of strains of an organism in the form of x/y/z: x. all (pathogenic and non-pathogenic)/, y. pathogenic/, z. non-pathogenic.

## Competing of interests

All authors declare that they have no competing interest.

## Authors’ contributions

VJ, RSC and CRT conceptualized and derived this study. VJ and CRT tested the models included in this study. VJ, SKC and AG designed and implemented the web server. VJ and CRT were involved in compilation of data sets. All authors have read and approved the final manuscript.

## Supplementary Material

Additional file 1: Table S1 Comparison of results for predicted protein vaccine candidate (PVC) by software, NERVE, and web servers, Vaxign, VaxiJen and Jenner-Predict from *Streptococcus pneumoniae* 70585 (gram positive) against experimentally known protective antigens. Click here for file

Additional file 2: Figure S1 Methodology followed in Jenner-Predict Web Server. Click here for file

Additional file 3: Table X1 Comparison of results for predicted protein vaccine candidate (PVC) by software, NERVE, and web servers, Vaxign, VaxiJen and Jenner-Predict from *Streptococcus pneumoniae* 70585 strain (gram positive). Click here for file

Additional file 4: Table X2 Comparison of results for predicted protein vaccine candidate (PVC) by software, NERVE, and web servers, Vaxign, VaxiJen and Jenner-Predict from Uropathogenic *Escherichia coli* strain CFT073 (gram negative). Click here for file

Additional file 5: Table S2 Comparison of results for predicted protein vaccine candidate (PVC) by software, NERVE, and web servers, Vaxign, VaxiJen and Jenner-Predict from *Escherichia coli* Uropathogenic strain CFT073 (gram positive) against experimentally known protective antigens. Click here for file

Additional file 6: Table S3 Results of protein vaccine candidate (PVC) prediction from vaccine candidate reported in Protegen database by software, NERVE, and web servers, Vaxign, VaxiJen and Jenner-Predict. Click here for file

Additional file 7: Table S4 Results of protein vaccine candidate (PVC) prediction from positive dataset used for VaxiJen server development by software, NERVE, and web servers, Vaxign, VaxiJen and Jenner-Predict. Click here for file
