# Supplementary material for: Jenner-predict server: prediction of protein vaccine candidates (PVCs) in bacteria based on host-pathogen interactions
Source: BMC Bioinformatics. 2013 Jul 1;14:211. doi: 10.1186/1471-2105-14-211 (PMC3701604; doi:10.1186/1471-2105-14-211)
Supplement: Additional file 1: Table S1 — Comparison of results for predicted protein vaccine candidate (PVC) by software, NERVE, and web servers, Vaxign, VaxiJen and Jenner-Predict from Streptococcus pneumoniae 70585 (gram positive) against experimentally known protective antigens. [file 1471-2105-14-211-S1.doc]

Table S1: Comparison of results for predicted protein vaccine candidate (PVC) by software, NERVE, and web servers, Vaxign, VaxiJen and Jenner-Predict from *Streptococcus pneumoniae* 70585 (gram positive) against experimentally known protective antigens^*^

| **^#^S. No.** | **Name of gene/protein** | **Gene ID** | **Localization** | **Nerve** | **Vaxign** | **VaxiJen** | **Jenner-Predict** | **Ref.** |
| --- | --- | --- | --- | --- | --- | --- | --- | --- |
| 1. | Pneumolysin (Thiol-activated cytolysin) | 225859688 | Extracellular | YES | NO | NO | YES | 1 |
| 2. | Pneumococcal choline binding protein A (PcpA) | 225859909 | Unknown | YES | YES | NO | YES | 2 |
| 3. | BVH-3  PhpA protein | 225858797 | Unknown | YES | NO | NO | NO | 3 |
| 4. | Autolysin lytA | 225859701 | Extracellular | YES | YES | NO | YES | 4 |
| 5. | Endo-beta-N-acetylglucosaminidase (SP046) | 225858758 | Extracellular | YES | YES | NO | YES | 5 |
| 6. | 1,4-beta-N-acetylmuramidase (SP091) | 225859330 | Extracellular | YES | YES | NO | YES | 5 |
| 7. | PspA | 225857997 | Extracellular | NO | YES | NO | YES | 6 |
| 8. | ABC transporter permease (Pit) | 225858856 | Cytoplasmic Membrane | NO | NO | NO | NO | 7 |
| 9. | Histidine triad protein B (SP036) | 225858962 | Non-Cytoplasmic | NO | NO | NO | NO | 5 |
| 10. | Putative protease maturation protein A (PpmA) | 225858774 | Cytoplasmic Membrane | NO | NO | YES | NO | 8 |
| 11. | PsaA | 225859406 | Cytoplasmic Membrane | NO | NO | NO | YES | 9 |
| 12. | Pneumococcal vaccine antigen A (SP101) | 225858817 | Cytoplasmic Membrane | NO | NO | NO | NO | 5 |
| 13. | Serine/threonine protein kinase (StkP) | 225859485 | Cytoplasmic Membrane | NO | NO | NO | YES | 10 |
| 14. | Pneumoniae neuraminidase (NanA) | 225859446 | Cellwall | NO | NO | YES | YES | 11 |
| 15. | CbpA or PspC or Hic or SpsA | 225858707 | Cytoplasmic Membrane | NO | NO | YES | NO | 12 |
| 16. | Zinc metalloprotease (ZmpB) | 225858492 | Cellwall | NO | NO | NO | NO | 13 |
| 17. | Endo-alpha-N-acetylgalactosaminidase | 225858223 | Cellwall | YES | YES | NO | YES | 14 |
| 18. | Pullulanase | 225858118 | Cellwall | NO | NO | NO | NO | 15 |

^*^ See details in materials and methods section. Jenner-Predict server is based on domains involved in host-pathogen interactions which are important in pathogenesis and disease establishment. For comparison with VaxiJen, a cut-off of 0.6 was used instead of default parameter 0.4 as it predicts almost half of proteome as vaccine candidates with default parameter.

^#^ S. No. indicates Serial Number; YES or NO denotes the corresponding protein is predicted or not-predicted, respectively by the corresponding software or web server.

**REFERENCES:**

1. Alexander JE, Lock RA, Peeters CC, Poolman JT, Andrew P W, Mitchell TJ, Ansman D, Paton JC: **Immunization of mice with pneumolysin toxoid confers a significant degree of protection against at least nine serotypes of *Streptococcus pneumoniae*.** *Infect Immun* 1994, **62**:5683–5688.

2. Glover DT, Hollingshead SK, Briles DE: ***Streptococcus pneumoniae* surface protein PcpA elicits protection against lung infection and fatal sepsis.** *Infect Immun* 2008, **76**:2767–2776.

3. Hamel J, Charland N, Pineau I, Ouellet C, Rioux S, Martin D, Brodeur BR: **Prevention of pneumococcal disease in mice immunized with conserved surface-accessible proteins.** *Infect Immun* 2004, **72**:2659–2670.

4. Berry AM, Lock RA, Hansman D, Paton JC: **Contribution of autolysin to virulence of *Streptococcus pneumoniae*.** *Infect Immun* 1989, **57**:2324–2330.

5. Wizemann TM, Heinrichs JH, Adamou JE, Erwin AL, Kunsch C, Choi GH, Barash SC, Rosen CA, Masure HR, et al: **Use of a whole genome approach to identify vaccine molecules affording protection against *Streptococcus pneumoniae* infection.** *Infect Immun* 2001, **69**:1593–1598.

6. Yamamoto M, McDaniel LS, Kawabata K, Briles DE, Jackson RJ, McGhee JR, Kiyono H: **Oral immunization with PspA elicits protective humoral immunity against *Streptococcus pneumoniae* infection.** *Infect. Immun* 1997, **65**:640–644.

7. Brown,J.S., Ogunniyi,A.D., Woodrow,M.C., Holden,D.W., and Paton,J.C. (2001) **Immunization with components of two iron uptake ABC transporters protects mice against systemic *Streptococcus pneumoniae* infection.** *Infect. Immun* 2001, **69**:6702–6706.

8. Overweg K, Kerr A, Sluijter M, Jackson MH, Mitchell TJ, de Jong AP, de Groot R, Hermans PW: **The putative proteinase maturation protein A of *Streptococcus pneumoniae* is a conserved surface protein with potential to elicit protective immune responses.** *Infect. Immun* 2000, **68**:4180–4188.

9. Briles DE, Ades E, Paton JC, Sampson JS, Carlone GM, Huebner RC, Virolainen A, Swiatlo E, Hollingshead SK: **Intranasal immunization of mice with a mixture of the pneumococcal proteins PsaA and PspA is highly protective against nasopharyngeal carriage of *Streptococcus pneumoniae*.** *Infect. Immun* 2000, **68**:796–800.

10. Giefing C, Meinke AL, Hanner M, Henics T, Bui MD, Gelbmann D, Lundberg U, Senn BM, Schunn M, Habel A, Henriques-Normark B, Ortqvist A, Kalin M, von G.abain A, Nagy E: **Discovery of a novel class of highly conserved vaccine antigens using genomic scale antigenic fingerprinting of pneumococcus with human antibodies.** *J Exp Med* 2008, **205**:117–131.

11. Tong HH, Li D, Chen S, Long JP, DeMaria TF: **Immunization with recombinant *Streptococcus pneumoniae* neuraminidase NanA protects chinchillas against nasopharyngeal colonization.** *Infect. Immun* 2005, **73**:7775–7778.

12. Ogunniyi AD, Woodrow MC, Poolman JT, Paton JC: **Protection against *Streptococcus pneumoniae* elicited by immunization with pneumolysin and CbpA.** *Infect Immun* 2001, **69**:5997–6003.

13. Gong Y, Xu W, Cui Y, Zhang X, Yao R, Li D, Wang H, He Y, Cao J, Yin Y: **Immunization with a ZmpB-based protein vaccine could protect against pneumococcal diseases in mice.** *Infect Immun* 2011, **79**:867– 878.

14. Caines ME, Zhu H, Vuckovic M, Willis LM, Withers SG, Wakarchuk WW, Strynadka NC: **The structural basis for T-antigen hydrolysis by Streptococcus pneumoniae: a target for structure-based vaccine design.** *J Biol Chem* 2008, **283**:31279–31283.

15. Bongaerts RJ, Heinz HP, Hadding U, Zysk G: **Antigenicity, expression, and molecular characterization of surface-located pullulanase of *Streptococcus pneumoniae*.** *Infect Immun* 2000, **68**:7141–7143.
