# Supplementary figures and images for: Jenner-predict server: prediction of protein vaccine candidates (PVCs) in bacteria based on host-pathogen interactions
Source: BMC Bioinformatics. 2013 Jul 1;14:211. doi: 10.1186/1471-2105-14-211 (PMC3701604; doi:10.1186/1471-2105-14-211)

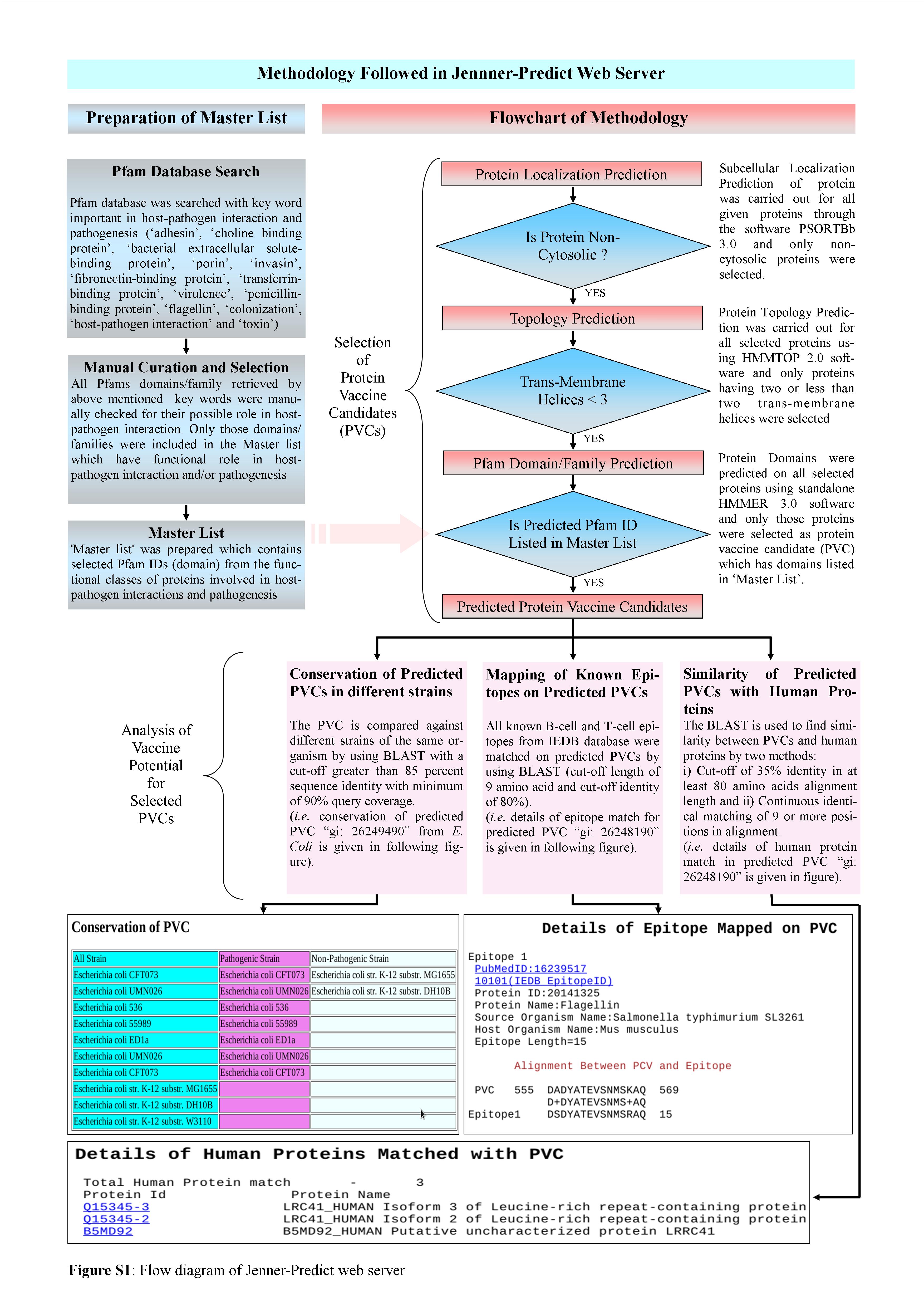

Supplement: Additional file 2: Figure S1 — Methodology followed in Jenner-Predict Web Server. [file 1471-2105-14-211-S2.jpeg]
