# Supplementary material for: Jenner-predict server: prediction of protein vaccine candidates (PVCs) in bacteria based on host-pathogen interactions
Source: BMC Bioinformatics. 2013 Jul 1;14:211. doi: 10.1186/1471-2105-14-211 (PMC3701604; doi:10.1186/1471-2105-14-211)
Supplement: Additional file 5: Table S2 — Comparison of results for predicted protein vaccine candidate (PVC) by software, NERVE, and web servers, Vaxign, VaxiJen and Jenner-Predict from Escherichia coli Uropathogenic strain CFT073 (gram positive) against experimentally known protective antigens. [file 1471-2105-14-211-S5.doc]

Table S2: Comparison of results for predicted protein vaccine candidate (PVC) by software, NERVE, and web servers, Vaxign, VaxiJen and Jenner-Predict from *Escherichia coli* Uropathogenic strain CFT073 (gram positive) against experimentally known protective antigens^*^

| **^#^S. No.** | **Locus** | **Gene Name** | **Gene ID**  **(GI)** | **Cellular Localization** | **NERVE** | **Vaxign** | **VaxiJen** | **Jenner-Predict** | **Ref.** |
| --- | --- | --- | --- | --- | --- | --- | --- | --- | --- |
| 1. | c0185 | FhuA | 26246096 | OuterMembrane | YES | YES | NO | YES | 1 |
| 2. | c0214 | YaeT | 26246123 | OuterMembrane | YES | NO | NO | YES | 1 |
| 3. | c0652 | OmpT | 26246544 | OuterMembrane | NO | NO | NO | NO | 1 |
| 4. | c0900 | OmpX | 26246790 | OuterMembrane | YES | YES | YES | YES | 1 |
| 5. | c1071 | OmpF | 26246956 | OuterMembrane | YES | NO | YES | YES | 1 |
| 6. | c1093 | OmpA | 26246978 | OuterMembrane | NO | NO | YES | YES | 1 |
| 7. | c1250 | IroN | 26247124 | OuterMembrane | YES | YES | YES | YES | 1 |
| 8. | c3655 | Ag43 | 26249490 | OuterMembrane | YES | YES | YES | YES | 1 |
| 9. | c1560 | NmpC | 26247429 | OuterMembrane | YES | YES | YES | YES | 1 |
| 10. | c1722 | OmpW | 26247587 | OuterMembrane | YES | YES | YES | YES | 1 |
| 11. | c2187 | YeaF | 26248041 | OuterMembrane | YES | YES | NO | NO | 1 |
| 12. | c2338 | FliC | 26248190 | Extracellular | YES | YES | YES | YES | 1 |
| 13. | c2482 | colicin | 26248334 | OuterMembrane | YES | YES | YES | YES | 1 |
| 14. | c2758 | OmpC | 26248604 | OuterMembrane | YES | YES | YES | YES | 1 |
| 15. | c3610 | Iha | 26249445 | OuterMembrane | YES | YES | YES | YES | 1 |
| 16. | c3623 | IutA | 26249458 | OuterMembrane | YES | YES | YES | YES | 1 |
| 17. | c3781 | TolC | 161486121 | OuterMembrane | NO | NO | NO | YES | 1 |
| 18. | c4095 | YheE | 26249919 | Cytoplasmic Membrane | NO | NO | YES | NO | 1 |
| 19. | c4308 | ChuA | 26250130 | OuterMembrane | YES | YES | YES | YES | 1 |
| 20. | c4894 | Tsx | 26250708 | OuterMembrane | YES | YES | YES | NO | 1 |
| 21. | c4929 | BtuB | 229106362 | OuterMembrane | NO | NO | NO | YES | 1 |
| 22. | c5006 | LamB | 26250818 | OuterMembrane | YES | YES | YES | YES | 1 |
| 23. | c5174 | IreA | 26250982 | OuterMembrane | NO | NO | YES | YES | 1 |
| 24. | c5400 | FimH | 26251208 | Unknown | YES | YES | YES | YES | 2 |
| 25. | c5188 | PapA | 26250996 | Extracellular | YES | YES | YES | YES | 3 |
| 26. | c3389 | __ | 26249224 | Cytoplasmic Membrane | NO | NO | NO | NO | 4 |
| 27. | c0393 | __ | 26246291 | Unknown | YES | YES | YES | YES | 4 |
| 28. | c4424 | __ | 26250246 | OuterMembrane | YES | YES | YES | YES | 4 |

^*^ See details in materials and methods section. Jenner-Predict server has been developed by us and is based on domains involved in host-pathogen interactions which are important in pathogenesis and disease establishment. For comparison with VaxiJen, a cut-off of 0.6 was used instead of default parameter 0.4 as it predicts almost half of proteome as vaccine candidates with default parameter.

^#^ S. No. indicates Serial Number; YES or NO denotes the corresponding protein is predicted or not-predicted, respectively by the corresponding software or web server.

**REFERENCES:**

1. Hagan EC, Mobley HL: **Uropathogenic *Escherichia coli* outer membrane antigens expressed during urinary tract infection.** *Infect. Immun* 2007, **75**:3941–3949.

2. Denich K, Blyn LB, Craiu A, Braaten BA, Hardy J, Low DA, O’Hanley PD: **DNA sequences of three papA genes from uropathogenic *Escherichia coli* strains: evidence of structural and serological conservation.** *Infect. Immun* 1991, **59**:3849–3858.

3. Ishikawa K, Normark S, Koenig S: **Vaccination with FimH adhesin protects cynomolgus monkeys from colonization and infection by uropathogenic *Escherichia coli.*** *J. Infect. Dis* 2000, **181**:774–778.

4. Durant L, Metais A, Soulama-Mouze C, Genevard JM, Nassif X, Escaich S: **Identification of candidates for a subunit vaccine against extra-intestinal pathogenic *Escherichia coli*.** *Infect Immun* 2007, **75**:1916–1925.
