# Supplementary material for: Jenner-predict server: prediction of protein vaccine candidates (PVCs) in bacteria based on host-pathogen interactions
Source: BMC Bioinformatics. 2013 Jul 1;14:211. doi: 10.1186/1471-2105-14-211 (PMC3701604; doi:10.1186/1471-2105-14-211)
Supplement: Additional file 6: Table S3 — Results of protein vaccine candidate (PVC) prediction from vaccine candidate reported in Protegen database by software, NERVE, and web servers, Vaxign, VaxiJen and Jenner-Predict. [file 1471-2105-14-211-S6.doc]

**Table S3**: Results of protein vaccine candidate (PVC) prediction from vaccine candidate reported in Protegen database by software, NERVE, and web servers, Vaxign, VaxiJen and Jenner-Predict^*^.

| **^#^S. No.** | **Protegen Database ID** | **Gene ID** | **Organism** | **Gram +/-** | **NERVE** | **Vaxign** | **VaxiJen** | **Jenner-Predict** | **^$^Pfam ID** | |
| --- | --- | --- | --- | --- | --- | --- | --- | --- | --- | --- |
|  | |360|VO: VO_0011020 | 52630374 | *Actinobacillus pleuropneumoniae* | n | YES | NO | NO | YES | PF02382 | |
|  | |362|VO: VO_0011022 | 190150285 | *Actinobacillus pleuropneumoniae* serovar 7 str. AP76 | n | NO | NO | YES | YES | PF08479 | |
|  | |38|VO: VO_0010873 | 47566484 | *Bacillus anthracis* str. 'Ames Ancestor | p | NO | NO | NO | YES | PF07737 | |
|  | |32|VO: VO_0010872 | 47566476 | *Bacillus anthracis* str. 'Ames Ancestor' | p | NO | NO | NO | YES | PF07691 | |
|  | |375|VO: VO_0011030 | 3980256 | *Bordetella pertussis* | n | YES | YES | YES | YES | PF05860 | |
|  | |376|VO: VO_0011031 | 580668 | *Bordetella pertussis* | n | NO | NO | YES | YES | PF03497 | |
|  | |377|VO: VO_0011032 | 225311181 | *Bordetella pertussis* | n | NO | NO | NO | YES | PF09275 | |
|  | |383|VO: VO_0011036 | 562026 | *Bordetella pertussis* | n | YES | NO | YES | YES | PF03212 | |
|  | |384|VO: VO_0011037 | 225311180 | *Bordetella pertussis* | n | YES | YES | NO | YES | PF03440 | |
|  | |385|VO: VO_0011038 | 225311183 | *Bordetella pertussis* | n | YES | NO | NO | YES | PF03440 | |
|  | |386|VO: VO_0011039 | 225311182 | *Bordetella pertussis* | n | YES | NO | NO | YES | PF09276 | |
|  | |378|VO: VO_0011033 | 33592195 | *Bordetella pertussis* Tohama I | n | NO | NO | YES | YES | PF03212 | |
|  | |379|VO: VO_0011034 | 33594638 | *Bordetella pertussis* Tohama I | n | NO | NO | NO | YES | PF02917 | |
|  | |838|VO: VO_0012384 | 11496927 | *Borrelia burgdorferi* B31 | n | YES | YES | YES | NO | PF00820 | |
|  | |839|VO: VO_0012385 | 11496910 | *Borrelia burgdorferi* B31 | n | NO | NO | YES | NO | PF00820 | |
|  | |840|VO: VO_0012386 | 11497024 | *Borrelia burgdorferi* B31 | n | YES | NO | YES | NO | PF01441 | |
|  | |228|VO: VO_0012365 | 62317941 | *Brucella abortus* bv. 1 str. 9-941 | n | NO | NO | YES | YES | PF01297 | |
|  | |110|VO: VO_0010948 | 122892474 | *Brucella melitensis* | n | NO | NO | NO | YES | PF01547 | |
|  | |232|VO: VO_0010962 | 17986819 | *Brucella melitensis* 16M | n | YES | NO | NO | NO | | PF04402 |
|  | |253|VO: VO_0010966 | 17987532 | *Brucella melitensis* 16M | n | YES | YES | YES | YES | PF01389 | |
|  | |254|VO: VO_0010967 | 17987867 | *Brucella melitensis* 16M | n | YES | YES | YES | YES | | PF06776 |
|  | |3|VO: VO_0010856 | 83269434 | *Brucella melitensis* biovar Abortus | n | NO | NO | YES | NO | PF00080 | |
|  | |682|VO: VO_0010908 | 82700077 | *Brucella melitensis* biovar Abortus 2308 | n | NO | NO | YES | NO | PF00542 | |
|  | |231|VO: VO_0010939 | 82700421 | *Brucella melitensis* biovar Abortus 2308 | n | YES | YES | YES | YES | PF01389 | |
|  | |258|VO: VO_0010971 | 82699574 | *Brucella melitensis* biovar Abortus 2308 | n | NO | NO | NO | YES | PF09312 | |
|  | |259|VO: VO_0010972 | 82700695 | *Brucella melitensis* biovar Abortus 2308 | n | YES | YES | YES | NO | PF02974 | |
|  | |260|VO: VO_0010973 | 82700483 | *Brucella melitensis* biovar Abortus 2308 | n | NO | NO | NO | YES | PF00691 | |
|  | |770|VO: VO_0011303 | 1929918 | *Burkholderia pseudomallei* | n | YES | YES | YES | YES | PF00669 | |
|  | |769|VO: VO_0010922 | 53721504 | *Burkholderia pseudomallei* K96243 | n | NO | NO | NO | YES | PF01547 | |
|  | |134|VO: VO_0010956 | 1813949 | *Campylobacter jejuni* | n | NO | NO | NO | YES | PF00497 | |
|  | |396|VO: VO_0011044 | 4704601 | *Campylobacter jejuni* | n | NO | YES | YES | YES | PF05736 | |
|  | |399|VO: VO_0011047 | 116292649 | *Campylobacter jejuni* | n | NO | YES | YES | NO | - | |
|  | |409|VO: VO_0011049 | 116292677 | *Campylobacter jejuni* | n | YES | NO | YES | NO | - | |
|  | |133|VO: VO_0010955 | 57237809 | *Campylobacter jejuni* RM1221 | n | NO | NO | NO | YES | PF00497 | |
|  | |398|VO: VO_0011046 | 121612545 | *Campylobacter jejuni* subsp. Jejuni 81-176 | n | NO | YES | YES | YES | PF00669 | |
|  | |400|VO: VO_0011048 | 121612344 | *Campylobacter jejuni* subsp. Jejuni 81-176 | n | NO | YES | NO | YES | PF00669 | |
|  | |87|VO: VO_0010942 | 15792662 | *Campylobacter jejuni* subsp. jejuni NCTC 11168 | n | YES | YES | YES | YES | | PF00669 |
|  | |397|VO: VO_0011045 | 112360246 | *Campylobacter jejuni* subsp. jejuni NCTC 11168 | n | YES | NO | NO | YES | PF00497 | |
|  | |435|VO: VO_0010890 | 1518659 | *Chlamydia muridarum* | n | YES | YES | YES | YES | PF01308 | |
|  | |430|VO: VO_0010885 | 15835130 | *Chlamydia muridarum* Nigg | n | YES | NO | NO | YES | | PF07244 |
|  | |432|VO: VO_0010887 | 15835057 | *Chlamydia muridarum* Nigg | n | NO | NO | NO | YES | PF04488 | |
|  | |433|VO: VO_0010888 | 15835381 | *Chlamydia muridarum* Nigg | n | NO | NO | NO | NO | PF01522 | |
|  | |434|VO: VO_0010889 | 15835382 | *Chlamydia muridarum* Nigg | n | NO | NO | NO | NO | - | |
|  | |436|VO: VO_0010891 | 15834883 | *Chlamydia muridarum* Nigg | n | YES | YES | NO | YES | | PF02415 |
|  | |438|VO: VO_0010893 | 15834882 | *Chlamydia muridarum* Nigg | n | YES | YES | NO | YES | PF07548 | |
|  | |425|VO: VO_0010881 | 40601 | *Chlamydophila abortus* | n | YES | YES | NO | YES | PF01308 | |
|  | |418|VO: VO_0011058 | 187438939 | *Chlamydophila abortus* | n | YES | YES | YES | YES | PF02415 | |
|  | |417|VO: VO_0011057 | 62184917 | *Chlamydophila abortus* S26/3 | n | NO | NO | NO | NO | PF02686 | |
|  | |419|VO: VO_0011059 | 62184696 | *Chlamydophila abortus* S26/3 | n | NO | NO | NO | YES | PF03717 | |
|  | |420|VO: VO_0011060 | 62184824 | *Chlamydophila abortus* S26/3 | n | YES | NO | YES | NO | PF03503 | |
|  | |428|VO: VO_0010883 | 15618244 | *Chlamydophila pneumoniae* | n | NO | YES | YES | YES | PF07201 | |
|  | |441|VO: VO_0010896 | 15618301 | *Chlamydophila pneumoniae* CWL029 | n | NO | NO | NO | NO | PF00436 | |
|  | |767|VO: VO_0010925 | 144545 | *Chlamydophila psittaci* | n | YES | YES | NO | YES | PF01308 | |
|  | |445|VO: VO_0010900 | 241183337 | *Clostridium botulinum* | p | NO | NO | NO | YES | PF01742 | |
|  | |449|VO: VO_0010904 | 169834607 | *Clostridium botulinum* B1 str. Okra | p | NO | NO | NO | YES | PF01742 | |
|  | |450|VO: VO_0010905 | 217781 | *Clostridium phage* c-st | p | NO | NO | NO | YES | PF01742 | |
|  | |451|VO: VO_0010909 | 157829735 | Clostridium tetani | p | NO | NO | NO | YES | PF07953 | |
|  | |750|VO: VO_0011287 | 38199106 | *Corynebacterium diphtheriae* | p | NO | NO | NO | YES | PF02763 | |
|  | |171|VO: VO_0010930 | 30025845 | *Coxiella burnetii* | n | YES | YES | YES | NO | - | |
|  | |225|VO: VO_0010938 | 9632507 | *Enterobacteria phage* 933W |  | YES | NO | YES | YES | | PF02258 |
|  | |92|VO: VO_0010943 | 157021162 | *Escherichia coli* | n | NO | YES | NO | YES | PF01376 | |
|  | |282|VO: VO_0010988 | 222104801 | *Escherichia coli* | n | NO | YES | YES | YES | PF07715 | |
|  | |79|VO: VO_0010941 | 110643341 | *Escherichia coli* 536 | n | NO | YES | NO | YES | PF04449 | |
|  | |240|VO: VO_0010964 | 157418230 | *Escherichia coli* APEC O1 | n | NO | NO | YES | YES | PF06291 | |
|  | |247|VO: VO_0010965| | 117624167 | *Escherichia coli* APEC O1 | n | NO | NO | YES | YES | PF07715 | |
|  | |278|VO: VO_0010984 | 26248334 | *Escherichia coli* CFT073 | n | YES | YES | YES | YES | PF07715 | |
|  | |279|VO: VO_0010985 | 26250982 | *Escherichia coli* CFT073 | n | NO | NO | YES | YES | PF07715 | |
|  | |280|VO: VO_0010986 | 26249458 | *Escherichia coli* CFT073 | n | YES | YES | YES | YES | PF07715 | |
|  | |283|VO: VO_0010989 | 26246291 | *Escherichia coli* CFT073 | n | YES | YES | YES | YES | PF02395 | |
|  | |284|VO: VO_0010990 | 26250246 | *Escherichia coli* CFT073 | n | YES | YES | YES | YES | PF05658 | |
|  | |199|VO: VO_0010940 | 15804222 | *Escherichia coli* O157:H7 EDL933 | n | NO | YES | YES | YES | PF07490 | |
|  | |93|VO: VO_0010944 | 15804220 | *Escherichia coli* O157:H7 EDL933 | n | NO | YES | YES | YES | PF01476 | |
|  | |289|VO: VO_0010993 | 209921909 | *Escherichia coli* SE11 | n | NO | YES | YES | YES | PF02432 | |
|  | |263|VO: VO_0010945 | 91213965 | *Escherichia coli* UTI89 | n | NO | NO | YES | YES | PF09160 | |
|  | |474|VO: VO_0011076 | 148688 | *Francisella tularensis* | n | YES | YES | YES | NO | - | |
|  | |476|VO: VO_0011078 | 115315051 | *Francisella tularensis* subsp. holarctica OSU18 | n | NO | YES | YES | YES | PF00691 | |
|  | |468|VO: VO_0011070 | 118496734 | *Francisella tularensis* subsp. novicida U112 | n | NO | YES | NO | YES | PF03938 | |
|  | |470|VO: VO_0011072 | 56708413 | *Francisella tularensis* subsp. tularensis SCHU S4 | n | YES | NO | YES | NO | PF11550 | |
|  | |475|VO: VO_0011077 | 56707247 | *Francisella tularensis* subsp. tularensis SCHU S4 | n | YES | NO | NO | NO | PF00081 | |
|  | |100|VO: VO_0010914 | 148896 | *Haemophilus influenzae* | n | YES | YES | NO | NO | PF03767 | |
|  | |102|VO: VO_0010916 | 21686508 | *Haemophilus influenzae* | n | YES | YES | YES | YES | | PF02395 |
|  | |103|VO: VO_0010917 | 23506944 | *Haemophilus influenzae* | n | YES | YES | YES | YES | | PF02395 |
|  | |276|VO: VO_0010918 | 4574246 | Haemophilus influenzae | n | NO | NO | YES | YES | PF03938 | |
|  | |824|VO: VO_0012376 | 2935168 | *Haemophilus influenzae* | n | NO | NO | NO | YES | PF00089 | |
|  | |480|VO: VO_0011081 | 148971 | *Haemophilus influenzae* | n | NO | NO | NO | YES | PF03009 | |
|  | |863|VO: VO_0012405 | 4929317 | *Haemophilus influenzae* | n | NO | NO | NO | YES | PF00149 | |
|  | |864|VO: VO_0012406 | 9716645 | *Haemophilus influenzae* | n | NO | YES | YES | YES | PF03349 | |
|  | |277|VO: VO_0010865 | 68249580 | *Haemophilus influenzae* 86-028NP | n | YES | YES | YES | YES | PF01298 | |
|  | |101|VO: VO_0010915 | 68248984 | *Haemophilus influenzae* 86-028NP | n | YES | YES | YES | YES | PF00691 | |
|  | |482|VO: VO_0011082 | 68249712 | *Haemophilus influenzae* 86-028NP | n | YES | NO | YES | YES | PF01389 | |
|  | |483|VO: VO_0011083 | 68249503 | *Haemophilus influenzae* 86-028NP | n | YES | YES | YES | YES | PF07244 | |
|  | |485|VO: VO_0011084 | 68248747 | *Haemophilus influenzae* 86-028NP | n | YES | YES | YES | YES | PF00267 | |
|  | |808|VO: VO_0011339 | 3128145 | *Helicobacter pylori* | n | NO | NO | NO | NO | PF00199 | |
|  | |810|VO: VO_0011341 | 50313203 | *Helicobacter pylori* | n | YES | YES | YES | YES | PF02691 | |
|  | |842|VO: VO_0012388 | 16802248 | *Listeria monocytogenes* | p | YES | NO | NO | YES | PF01289 | |
|  | |862|VO: VO_0012404 | 215422507 | *Listeria monocytogenes* | p | YES | YES | YES | NO | - | |
|  | |813|VO: VO_0011344 | 26513901 | *Listonella anguillarum* | n | YES | NO | YES | YES | PF00267 | |
|  | |814|VO: VO_0012366 | 224992215 | *Mycobacterium bovis* BCG str. Tokyo 172 | p | NO | YES | NO | YES | PF00756 | |
|  | |154|VO: VO_0010936 | 29027587 | *Mycobacterium tuberculosis* | n | NO | YES | NO | YES | PF00756 | |
|  | |821|VO: VO_0012373 | 148660242 | *Mycobacterium tuberculosis* H37Ra | p | NO | NO | YES | NO | - | |
|  | |104|VO: VO_0010919 | 57117165 | *Mycobacterium tuberculosis* H37Rv | p | Yes 2 | YES | 0.5657 | YES | PF06013 | |
|  | |118|VO: VO_0012364 | 15609023 | *Mycobacterium tuberculosis* H37Rv | p | YES | YES | NO | YES | | PF00756 |
|  | |121|VO: VO_0010951 | 15611010 | *Mycobacterium tuberculosis* H37Rv | p | YES | YES | YES | YES | | PF06013 |
|  | |123|VO: VO_0010953 | 15607267 | *Mycobacterium tuberculosis* H37Rv | p | NO | NO | YES | YES | PF00089 | |
|  | |815|VO: VO_0012367 | 15609117 | *Mycobacterium tuberculosis* H37Rv | p | YES | YES | NO | NO | PF11738 | |
|  | |818|VO: VO_0012370 | 57116798 | *Mycobacterium tuberculosis* H37Rv | p | YES | YES | YES | YES | PF01547 | |
|  | |819|VO: VO_0012371 | 57116919 | *Mycobacterium tuberculosis* H37Rv | p | YES | YES | NO | NO | PF00934 | |
|  | |820|VO: VO_0012372 | 57116920 | *Mycobacterium tuberculosis* H37Rv | p | NO | NO | NO | YES | PF00823 | |
|  | |588|VO: VO_0011175 | 57116926 | *Mycobacterium tuberculosis* H37Rv | p | YES | YES | NO | YES | PF07174 | |
|  | |589|VO: VO_0011176 | 15609063 | *Mycobacterium tuberculosis* H37Rv | p | YES | NO | YES | YES | PF09167 | |
|  | |865|VO: VO_0012407 | 57116801 | *Mycobacterium tuberculosis* H37Rv | p | YES | YES | YES | YES | PF01547 | |
|  | |867|VO: VO_0012409 | 15610010 | *Mycobacterium tuberculosis* H37Rv | p | NO | NO | NO | YES | PF02469 | |
|  | |591|VO: VO_0011178 | 7249262 | *Mycoplasma gallisepticum* | n | YES | YES | NO | YES | PF07554 | |
|  | |833|VO: VO_0012380 | 8926211 | *Neisseria meningitidis* | n | YES | YES | YES | YES | PF07715 | |
|  | |597|VO: VO_0011182 | 1017433 | *Neisseria meningitidis* | n | YES | YES | YES | YES | PF01298 | |
|  | |264|VO: VO_0010946 | 15677945 | *Neisseria meningitidis* MC58 | n | YES | NO | YES | YES | PF01298 | |
|  | |265|VO: VO_0010947 | 15677829 | *Neisseria meningitidis* MC58 | n | NO | NO | NO | YES | PF00877 | |
|  | |266|VO: VO_0010974 | 15677776 | *Neisseria meningitidis* MC58 | n | n | NO | NO | YES | PF03180 | |
|  | |268|VO: VO_0010976 | 15677037 | *Neisseria meningitidis* MC58 | n | YES | YES | NO | NO | PF05643 | |
|  | |269|VO: VO_0010977 | 15676883 | *Neisseria meningitidis* MC58 | n | YES | YES | YES | YES | PF03895 | |
|  | |270|VO: VO_0010978 | 15675973 | *Neisseria meningitidis* MC58 | n | YES | YES | NO | NO | PF03562 | |
|  | |271|VO: VO_0010979 | 15676561 | *Neisseria meningitidis* MC58 | n | YES | YES | YES | YES | PF02462 | |
|  | |272|VO: VO_0010980 | 15677822 | *Neisseria meningitidis* MC58 | n | NO | YES | YES | YES | | PF03895 |
|  | |273|VO: VO_0010981 | 15677705 | *Neisseria meningitidis* MC58 | n | YES | YES | YES | NO | | PF08794 |
|  | |274|VO: VO_0010982 | 15677911 | *Neisseria meningitidis* MC58 | n | YES | YES | NO | NO | PF04972 | |
|  | |275|VO: VO_0010983 | 15676917 | *Neisseria meningitidis* MC58 | n | YES | YES | YES | NO | PF04264 | |
|  | |594|VO: VO_0011180 | 15676020 | *Neisseria meningitidis* MC58 | n | YES | YES | YES | YES | PF03349 | |
|  | |843|VO: VO_0012360 | 15596974 | *Pseudomonas aeruginosa* PAO1 | n | YES | YES | YES | YES | PF05736 | |
|  | |844|VO: VO_0012361 | 15596903 | *Pseudomonas aeruginosa* PAO1 | n | NO | NO | NO | YES | PF04792 | |
|  | |845|VO: VO_0012362 | 15598049 | *Pseudomonas aeruginosa* PAO1 | n | YES | NO | YES | NO | PF11839 | |
|  | |786|VO: VO_0011317 | 152498 | *Rickettsia prowazekii* | n | YES | YES | YES | YES | PF12334 | |
|  | |650|VO: VO_0011234 | 112710 | *Rickettsia rickettsii* | n | NO | YES | YES | YES | PF03797 | |
|  | |651|VO: VO_0011235 | 6685726 | *Rickettsia rickettsii* | n | NO | YES | YES | YES | PF12334 | |
|  | |318|VO: VO_0010997 | 259475459 | *Salmonella enterica* subsp. enterica serovar Typhimurium | n | YES | YES | YES | YES | PF07715 | |
|  | |321|VO: VO_0010999 | 54036439 | *Salmonella enterica* subsp. enterica serovar Typhimurium | n | NO | NO | NO | YES | PF05925 | |
|  | |307|VO: VO_0010994 | 22036246 | *Shigella flexneri* 2a | n | YES | YES | NO | YES | PF04888 | |
|  | |308|VO: VO_0010995 | 47056 | *Shigella flexneri* 2a | n | YES | NO | YES | YES | PF09599 | |
|  | |309|VO: VO_0010996 | 22036352 | *Shigella flexneri* 2a | n | YES | NO | NO | YES | PF06511 | |
|  | |288|VO: VO_0010992 | 13449098 | *Shigella flexneri* 5a | n | YES | NO | YES | NO | - | |
|  | |849|VO: VO_0012392 | 120457 | *Staphylococcus aureus* subsp NCTC 8325 | p | NO | YES | YES | YES | PF04650 | |
|  | |848|VO: VO_0012391 | 49482291 | *Staphylococcus aureus* subsp. aureus MRSA252 | p | NO | NO | NO | YES | PF05223 | |
|  | |847|VO: VO_0012390 | 151220968 | *Staphylococcus aureus* subsp. aureus str. Newman | p | NO | NO | YES | YES | PF04650 | |
|  | |850|VO: VO_0012393 | 5327234 | *Streptococcus agalactiae* | p | YES | YES | YES | YES | PF04650 | |
|  | |860|VO: VO_0012403 | 1620648 | *Streptococcus agalactiae* | p | NO | NO | YES | YES | | PF04650 |
|  | |859|VO: VO_0012402 | 76788047 | *Streptococcus agalactiae* A909 | p | YES | YES | YES | YES | PF01476 | |
|  | |627|VO: VO_0011211 | 225870123 | *Streptococcus equi* subsp. equi 4047 | p | NO | YES | NO | YES | PF01468 | |
|  | |629|VO: VO_0011213 | 225870316 | *Streptococcus equi* subsp. equi 4047 | p | NO | YES | YES | YES | PF05737 | |
|  | |631|VO: VO_0011215 | 225869898 | *Streptococcus equi* subsp. equi 4047 | p | NO | NO | YES | NO | - | |
|  | |632|VO: VO_0011216 | 225871286 | *Streptococcus equi* subsp. equi 4047 | p | NO | NO | NO | YES | PF04650 | |
|  | |630|VO: VO_0011214 | 225869227 | *Streptococcus equi* subsp. zooepidemicus | p | NO | YES | YES | YES | PF08341 | |
|  | |605|VO: VO_0011190 | 209867628 | *Streptococcus pneumoniae* | p | NO | NO | NO | YES | PF01297 | |
|  | |607|VO: VO_0011192 | 116515376 | *Streptococcus pneumoniae* D39 | p | NO | NO | NO | YES | PF01289 | |
|  | |619|VO: VO_0011203 | 116515359 | *Streptococcus pneumoniae* D39 | p | NO | YES | YES | YES | PF04650 | |
|  | |620|VO: VO_0011204 | 116515876 | *Streptococcus pneumoniae* D39 | p | NO | YES | NO | YES | PF01473 | |
|  | |853|VO: VO_0012396 | 14915682 | *Streptococcus pyogenes* | p | NO | NO | YES | YES | PF04650 | |
|  | |855|VO: VO_0012398 | 90567992 | *Streptococcus pyogenes* serotype M12 | p | YES | YES | NO | NO | PF09028 | |
|  | |446|VO: VO_0010901 | 166236883 | synthetic construct | p | YES | YES | NO | YES | | PF07953 |
|  | |584|VO: VO_0011173 | 283801990 | synthetic construct | s | YES | YES | YES | YES | PF00595 | |
|  | |856|VO: VO_0012399 | 15639249 | *Treponema pallidum* subsp. pallidum str. Nichols | n | NO | NO | NO | YES | PF03009 | |
|  | |857|VO: VO_0012400 | 15639756 | *Treponema pallidum* subsp. pallidum str. Nichols | n | NO | NO | YES | NO | - | |
|  | |858|VO: VO_0012401 | 159158965 | *Treponema pallidum* subsp. pallidum str. Nichols | n | YES | YES | YES | YES | PF01103 | |
|  | |731|VO: VO_0011270 | 110264635 | *Vibrio cholerae* O1 | n | NO | NO | YES | YES | PF01376 | |
|  | |732|VO: VO_0011271 | 21616882 | *Vibrio cholerae* O1 | n | NO | YES | NO | YES | PF05946 | |
|  | |733|VO: VO_0011272 | 15640854 | *Vibrio cholerae* O1 biovar El Tor str. N16961 | n | YES | YES | YES | YES | | PF06340 |
|  | |340|VO: VO_0011003 | 162417777 | *Yersinia pestis* Angola | n | NO | NO | NO | NO | PF00069 | |
|  | |23|VO: VO_0010870 | 45478667 | *Yersinia pestis* biovar Microtus str. 91001] | n | YES | YES | YES | NO | PF09255 | |
|  | |44|VO: VO_0010874 | 16082719 | *Yersinia pestis* CO92 | n | NO | NO | NO | NO | PF04792 | |
|  | |48|VO: VO_0010877 | 16082686 | *Yersinia pestis* CO92 | n | YES | YES | YES | NO | PF01278 | |
|  | |49|VO: VO_0010878 | 16082716 | *Yersinia pestis* CO92 | n | NO | NO | NO | YES | PF05844 | |
|  | |51|VO: VO_0012359 | 16082755 | *Yersinia pestis* CO92 | n | NO | NO | YES | YES | PF09013 | |
|  | |54|VO: VO_0010913 | 16082743 | *Yersinia pestis* CO92 | n | NO | NO | NO | YES | PF09392 | |
|  | |342|VO: VO_0011005 | 218927800 | *Yersinia pestis* CO92 | n | NO | YES | YES | YES | PF03797 | |
|  | |344|VO: VO_0011007 | 218927806 | *Yersinia pestis* CO92 | n | NO | NO | NO | YES | PF01547 | |
|  | |346|VO: VO_0011009 | 218930092 | *Yersinia pestis* CO92 | n | NO | NO | NO | NO | PF00884 | |
|  | |347|VO: VO_0011010 | 218928525 | *Yersinia pestis* CO92 | n | NO | NO | NO | NO | PF03548 | |
|  | |349|VO: VO_0011012 | 218927621 | *Yersinia pestis* CO92 | n | NO | NO | YES | NO | - | |
|  | |350|VO: VO_0011013 | 218930726 | *Yersinia pestis* CO92 | n | NO | NO | NO | YES | PF08479 | |

^*^ For details, see materials and methods section. Jenner-Predict server is based on domains involved in host-pathogen interactions which are important in pathogenesis and disease establishment. Out of total reported 257 bacterial protective PVCs reported in the Protegen database, 177 bacterial protective antigens having less than 90 percent identity were selected for evaluation purpose from 200 proteins with non-cytosolic cellular localization and having less than two transmembrane helices. For comparison with VaxiJen, a cut-off of 0.6 was used instead of default parameter 0.4 as it predicts almost half of proteome as vaccine candidates with default parameter.

^#^ S. No. indicates Serial Number; p or n in Gram column indicate gram positive and gram negative, respectively; and YES or NO denotes the corresponding protein is predicted or not-predicted, respectively by the corresponding software or web server.

^$^ Pfam ID can be used to find domains and associated functions of proteins from Pfam database (http://pfam.janelia.org/).
