# Supplementary material for: Jenner-predict server: prediction of protein vaccine candidates (PVCs) in bacteria based on host-pathogen interactions
Source: BMC Bioinformatics. 2013 Jul 1;14:211. doi: 10.1186/1471-2105-14-211 (PMC3701604; doi:10.1186/1471-2105-14-211)
Supplement: Additional file 7: Table S4 — Results of protein vaccine candidate (PVC) prediction from positive dataset used for VaxiJen server development by software, NERVE, and web servers, Vaxign, VaxiJen and Jenner-Predict. [file 1471-2105-14-211-S7.doc]

**Table S4 (A)**: Results of protein vaccine candidate (PVC) prediction from positive dataset used for VaxiJen server development by software, NERVE, and web servers, Vaxign, VaxiJen and Jenner-Predict^*^.

| **^#^S. No.** | **SwissProt ID** | **Gram** | **Localization** | **Organism** | **Nerve** | **VaxiJen** | **Vaxign** | **Jenner-Predict** |
| --- | --- | --- | --- | --- | --- | --- | --- | --- |
| 1. | Q9RN24 | P | Extracellular | *Bacillus anthracis* | YES | YES | YES | YES |
| 2. | Q93V22  P04977 | N | Extracellular | *Bordetella pertussis* | NO | NO | NO | YES |
| 3. | Q9S6N1 | N | Outer Membrane | *Bordetella pertussis* | NO | YES | NO | YES |
| 4. | P14013  P0C926 | N |  | *Borrelia burgdorferi* | YES | YES | NO | NO |
| 5. | P17739 | N | Outer Membrane | *Borrelia burgdorferi* | YES | YES | YES | NO |
| 6. | P70854 | N | Unknown | *Borrelia burgdorferi* | NO | NO | NO | YES |
| 7. | Q07337 | N | Outer Membrane | *Borrelia burgdorferi* | YES | YES | NO | NO |
| 8. | P0A470 | N | Unknown | *Brucella abortus* | NO | NO | NO | NO |
| 9. | P15453 | N | Periplasmic | *Brucella abortus* | YES | YES | NO | NO |
| 10. | Q45321 | N | Outer Membrane | *Brucella melitensis* | YES | YES | YES | YES |
| 11. | P27053 | N | Extracellular | *Campylobacter coli* | YES | YES | YES | YES |
| 12. | Q46412 | N | Outer Membrane | *Chlamydia trachomatis* | YES | YES | YES | YES |
| 13. | Q9RF12 | P | Extracellular | *Clostridium perfringens* | YES | YES | YES | NO |
| 14. | Q9RM68 | P | Unknown | *Clostridium perfringens* | YES | YES | NO | YES |
| 15. | Q9LA13 | P | Extracellular | *Clostridium tetani* | NO | NO | NO | YES |
| 16. | P20626 | P | Unknown | *Corynebacterium pseudotuberculosis* | YES | NO | YES | NO |
| 17. | P05825 | N | Outer Membrane | *Escherichia coli* | YES | YES | YES | YES |
| 18. | P08191 | N | Unknown | *Escherichia coli* | YES | YES | YES | YES |
| 19. | P0AFZ6 | N | Cytoplasmic Membrane | *Escherichia coli* | NO | NO | NO | NO |
| 20. | P17315 | N | Outer Membrane | *Escherichia coli* | YES | YES | NO | YES |
| 21. | Q93V32 | N | Unknown | *Escherichia coli* | YES | NO | YES | YES |
| 22. | P43838 | N | Outer Membrane | *Haemophilus influenzae* | YES | YES | YES | YES |
| 23. | P10324 | N | Outer Membrane | *Haemophilus influenzae* | YES | YES | YES | YES |
| 24. | P45996 | N | Outer Membrane | *Haemophilus influenzae* | YES | YES | NO | YES |
| 25. | Q9ZKX5 | N | Unknown | *Helicobacter pylori* | NO | NO | NO | NO |
| 26. | P24017 | N | Outer Membrane | *Klebsiella pneumoniae* | YES | YES | NO | YES |
| 27. | Q48427 | N | Outer Membrane | *Klebsiella pneumoniae* | YES | YES | YES | YES |
| 28. | Q48473 | N | Outer Membrane | *Klebsiella pneumoniae* | YES | YES | NO | YES |
| 29. | P21347 | N | Extracellular | *Klebsiella pneumoniae* | YES | YES | YES | NO |
| 30. | Q9Z374 | N | Unknown | *Klebsiella pneumoniae* | YES | NO | YES | NO |
| 31. | P21171 | P | Extracellular | *Listeria monocytogenes* | YES | YES | YES | YES |
| 32. | Q9L5B9 | P | Extracellular | *Listeria monocytogenes* | YES | NO | NO | YES |
| 33. | Q06947 | P | Extracellular | *Mycobacterium avium* | YES | YES | YES | YES |
| 34. | P0A4V3  P0C926 | N | Outermembrane | *Mycobacterium bovis* | YES | YES | NO | NO |
| 35. | P0A671 | P | Cytoplasmic Membrane | *Mycobacterium bovis* | NO | NO | NO | YES |
| 36. | O05870 | P | Extracellular | *Mycobacterium tuberculosis* | YES | YES | YES | YES |
| 37. | P0A564 | P | Extracellular | *Mycobacterium tuberculosis* | YES | YES | YES | YES |
| 38. | P0A5P6 | P | Cellwall | *Mycobacterium tuberculosis* | NO | NO | YES | NO |
| 39. | P0A5Q2 | P | Extracellular | *Mycobacterium tuberculosis* | YES | YES | NO | YES |
| 40. | P0A5Q4 | P | Extracellular | *Mycobacterium tuberculosis* | YES | YES | YES | NO |
| 41. | P0A5Y2 | P | Unknown | *Mycobacterium tuberculosis* | YES | YES | YES | YES |
| 42. | P0A670 | P | Cytoplasmic Membrane | *Mycobacterium tuberculosis* | NO | NO | YES | YES |
| 43. | P31952  A5U3Q3 | P | Extracellular | *Mycobacterium tuberculosis* | YES | YES | NO | YES |
| 44. | Q79F92 | P | Cytoplasmic Membrane | *Mycobacterium tuberculosis* | NO | NO | NO | NO |
| 45. | O07175 | P | Unknown | *Mycobacterium tuberculosis* | NO | YES | NO | YES |
| 46. | O50430 | P | Cytoplasmic Membrane | *Mycobacterium tuberculosis* | NO | NO | YES | NO |
| 47. | P0A4V6 | P | Extracellular | *Mycobacterium tuberculosis* | YES | YES | YES | YES |
| 48. | P0A566 | P | Extracellular | *Mycobacterium tuberculosis* | YES | YES |  | YES |
| 49. | P0A568 | P | Unknown | *Mycobacterium tuberculosis* | YES | YES | YES | YES |
| 50. | P0A5B7 | P | Cellwall | *Mycobacterium tuberculosis* | YES | YES | NO | NO |
| 51. | P0A5P2 | P | Extracellular | *Mycobacterium tuberculosis* | NO | YES | NO | NO |
| 52. | P0A5P8 | P | Extracellular | *Mycobacterium tuberculosis* | YES | YES | YES | NO |
| 53. | P0A5Y2 | P | Unknown | *Mycobacterium tuberculosis* | YES | YES | YES | YES |
| 54. | P15712 | P | Unknown | *Mycobacterium tuberculosis* | YES | YES | YES | YES |
| 55. | P65306 | P | Cytoplasmic Membrane | *Mycobacterium tuberculosis* | NO | NO | NO | NO |
| 56. | Q7D8M9 | P | Cytoplasmic Membrane | *Mycobacterium tuberculosis* | NO | NO | YES | YES |
| 57. | P96943 | N | Outer Membrane | *Neisseria meningitidis* | YES | YES | YES | YES |
| 58. | Q53348 | N | Outer Membrane | *Neisseria meningitidis* | YES | YES | YES | YES |
| 59. | Q53990 | N | Extracellular | *Neisseria meningitidis* | YES | YES | NO | YES |
| 60. | O30527 | N | Extracellular | *Pseudomonas aeruginosa* | NO | NO | NO | YES |
| 61. | P11439 | N | Extracellular | *Pseudomonas aeruginosa* | NO | NO | NO | YES |
| 62. | 32722 | N | Outer Membrane | *Pseudomonas aeruginosa* | NO | YES | YES | YES |
| 63. | P13794 | N | Outer Membrane | *Pseudomonas aeruginosa* | YES | YES | YES | YES |
| 64. | Q8ZP50 | N | Outer Membrane | *Salmonella typhimurium* | YES | YES | YES | YES |
| 65. | P69178 | N | Unknown | *Shigella dysenteriae* | YES | YES | NO | YES |
| 66. | P0A0L2 | P | Extracellular | *Staphylococcus aureus* | YES | YES | NO | YES |
| 67. | Q53653 | P | Cellwall | *Staphylococcus aureus* | YES | YES | NO | YES |
| 68. | Q3K3Z5 | P | Extracellular | *Streptococcus agalactiae* | YES | YES | YES | YES |
| 69. | Q9ZHG7 | P | Unknown | *Streptococcus agalactiae* | NO | NO | NO | NO |
| 70. | O34097 | P | Extracellular | *Streptococcus pneumoniae* | NO | YES | YES | YES |
| 71. | P11990  P0C2J9 | P | Extracellular | *Streptococcus pneumoniae* | YES | NO | NO | YES |
| 72. | Q8DN05 | P | Extracellular | *Streptococcus pneumoniae* | YES | YES | YES | YES |
| 73. | Q8VQ82 | P | Cytoplasmic Membrane | *Streptococcus pneumoniae* | NO | NO | YES | YES |
| 74. | Q9AG74 | P | Unknown | *Streptococcus pneumoniae* | NO | NO | NO | NO |
| 75. | P59206 | P | Extracellular | *Streptococcus pneumoniae* | YES | NO | YES | YES |
| 76. | Q9Z4J8 | P | Unknown | *Streptococcus pneumoniae* | YES | NO | YES | YES |
| 77. | O30405 | N | Outer Membrane | *Treponema pallidum* | NO | NO | NO | YES |
| 78. | O83867 | N | Unknown | *Treponema pallidum* | NO | YES | NO | YES |
| 79. | P19649 | N | Outer Membrane | *Treponema pallidum* | NO | YES | NO | NO |
| 80. | Q87L97 | N | Cytoplasmic Membrane | *Vibrio parahaemolyticus* | NO | NO | NO | NO |
| 81. | P21206  A4TSQ1 | N | Extracellular | *Yersinia pestis* | NO | NO | NO | YES |
| 82. | P26948 | N | Extracellular | *Yersinia pestis* | YES | YES | YES | NO |
| 83. | Q7DHH4 | P | Cytoplasmic Membrane | *Staphylococcus aureus* | NO | NO | NO | YES |

^*^ See details in materials and methods section. Jenner-Predict server has been developed by us and is based on domains involved in host-pathogen interactions which are important in pathogenesis and disease establishment. Out of total 100 bacterial protective antigen in positive dataset used for VaxiJen server, 83 proteins with non-cytosolic cellular localization and having less than two transmembrane helices were selected evaluation by different methods. For VaxiJen, a cut-off of 0.6 was used instead of default parameter 0.4 as it predicts almost half of a bacterial proteome as vaccine candidates with default parameter.

^#^ S. No. indicates Serial Number; P or N in Gram column indicate gram positive and gram negative, respectively; and YES or NO denotes the corresponding protein is predicted or not-predicted, respectively by the corresponding software or web server.

**Table S4 (B)**: Results of protein vaccine candidate (PVC) prediction from negative dataset used for VaxiJen server development by software, NERVE, and web servers, Vaxign, VaxiJen and Jenner-Predict^*^

| **^#^S. No.** | **SwissProt ID** | **Organism** | **Gram** | **Nerve** | **VaxiJen** | **Vaxign** | **Jenner-Predict** |
| --- | --- | --- | --- | --- | --- | --- | --- |
| 1. | P26826 | *Clostridium perfringens* | P | YES | NOT | YES | NOT |
| 2. | Q8XMI8 | *Clostridium perfringens* | P | NOT | NOT | NOT | NOT |
| 3. | Q890Y8 | *Clostridium tetani* | P | NOT | NOT | NOT | NOT |
| 4. | Q8Y652 | *Listeria monocytogenes* | P | NOT | NOT | NOT | NOT |
| 5. | Q48909 | *Mycobacterium avium* | P | NOT | NOT | NOT | NOT |
| 6. | Q48919 | *Mycobacterium avium* | P | YES | NOT | YES | YES |
| 7. | Q7TXM7 | *Mycobacterium bovis* | P | NOT | YES | NOT | NOT |
| 8. | O69742 | *Mycobacterium tuberculosis* | P | YES | YES | YES | NOT |
| 9. | O69743 | *Mycobacterium tuberculosis* | P | NOT | NOT | NOT | NOT |
| 10. | P0A5R6 | *Mycobacterium tuberculosis* | P | NOT | NOT | NOT | NOT |
| 11. | P96910 | *Mycobacterium tuberculosis* | P | NOT | NOT | NOT | NOT |
| 12. | Q79F93 | *Mycobacterium tuberculosis* | P | YES | NOT | NOT | NOT |
| 13. | P0A5N0 | *Mycobacterium tuberculosis* | P | NOT | NOT | NOT | NOT |
| 14. | P63338 | *Mycobacterium tuberculosis* | P | NOT | NOT | NOT | NOT |
| 15. | P64249 | *Mycobacterium tuberculosis* | P | NOT | NOT | NOT | NOT |
| 16. | Q8ZIC6 | *Yersinia pestis* | N | NOT | NOT | NOT | NOT |
| 17. | O07341 | *Streptococcus pneumoniae* | P | NOT | NOT | NOT | NOT |
| 18. | O33754 | *Streptococcus pneumoniae* | P | NOT | NOT | NOT | NOT |
| 19. | Q7VWV9 | *Bordetella pertussis* | N | NOT | NOT | NOT | NOT |
| 20. | O51043 | *Borrelia burgdorferi* | N | NOT | NOT | NOT | NOT |
| 21. | O51240 | *Borrelia burgdorferi* | N | NOT | NOT | NOT | NOT |
| 22. | Q05051 | *Borrelia burgdorferi* | N | YES | NOT | YES | NOT |
| 23. | P0AD79 | *Escherichia coli* | N | NOT | NOT | NOT | NOT |
| 24. | O25798 | *Helicobacter pylori* | N | YES | NOT | NOT | NOT |
| 25. | Q3S3S0 | *Helicobacter pylori* | N | NOT | NOT | NOT | NOT |
| 26. | P20440 | *Klebsiella pneumoniae* | N | YES | NOT | NOT | NOT |
| 27. | Q48439 | *Klebsiella pneumoniae* | N | NOT | NOT | NOT | NOT |
| 28. | Q84HD6 | *Neisseria meningitidis* | N | NOT | NOT | NOT | NOT |
| 29. | P23181 | *Pseudomonas aeruginosa* | N | NOT | NOT | NOT | NOT |
| 30. | Q53247 | *Orientia tsutsugamushi* | N | NOT | NOT | NOT | YES |
| 31. | P07643 | *reponema pallidum* | N | NOT | YES | NOT | NOT |
| 32. | P29724 | *Treponema pallidum* | N | NOT | NOT | NOT | NOT |
| 33. | Q8ZF61 | *Yersinia pestis* | N | YES | NOT | YES | NOT |

^*^ See details in materials and methods section. Jenner-Predict server is based on domains involved in host-pathogen interactions which are important in pathogenesis and disease establishment. Out of total 100 bacterial protective antigen in negative dataset used for VaxiJen server, 34 proteins with non-cytosolic cellular localization and having less than two transmembrane helices were selected evaluation by different methods. For VaxiJen, a cut-off of 0.6 was used instead of default parameter 0.4 as it predicts almost half of a bacterial proteome as vaccine candidates with default parameter.

^#^ S. No. indicates Serial Number; P or N in Gram column indicate gram positive and gram negative, respectively; and YES or NO denotes the corresponding protein is predicted or not-predicted, respectively by the corresponding software or web server.
